# Streamlined, single-step non-viral CRISPR-Cas9 knockout strategy enhances gene editing efficiency in primary human chondrocyte populations

**DOI:** 10.1186/s13075-024-03294-w

**Published:** 2024-03-11

**Authors:** Simone Ponta, Angela Bonato, Philipp Neidenbach, Valentino F. Bruhin, Alexis Laurent, Lee Ann Applegate, Marcy Zenobi-Wong, Goncalo Barreto

**Affiliations:** 1 Department of Health Sciences and Technology, ETH Zürich, Zurich, 8093 Switzerland; 2 Schulthess Clinic, Department of Lower Extremity Orthopaedics, Musculoskeletal Centre, Zurich, 8008 Switzerland; 3 Regenerative Therapy Unit, Plastic, Reconstructive & Hand Surgery Service, Lausanne University Hospital, University of Lausanne, Epalinges, 1066 Switzerland; 4 Clinicum, Faculty of Medicine, University of Helsinki and Helsinki University Hospital, Helsinki, 00014 Finland; 5 Medical Ultrasonics Laboratory (MEDUSA), Department of Neuroscience and Biomedical Engineering, Aalto University, Espoo, 02150 Finland; 6 Orton Orthopedic Hospital, Tenholantie 10, Helsinki, 00280 Finland

**Keywords:** Gene editing, CRISPR-Cas9, Primary chondrocytes, NF-κB, RELA

## Abstract

**Background:**

CRISPR-Cas9-based genome engineering represents a powerful therapeutic tool for cartilage tissue engineering and for understanding molecular pathways driving cartilage diseases. However, primary chondrocytes are difficult to transfect and rapidly dedifferentiate during monolayer (2D) cell culture, making the lengthy expansion of a single-cell-derived edited clonal population not feasible. For this reason, functional genetics studies focused on cartilage and rheumatic diseases have long been carried out in cellular models that poorly recapitulate the native molecular properties of human cartilaginous tissue (e.g., cell lines, induced pluripotent stem cells). Here, we set out to develop a non-viral CRISPR-Cas9, bulk-gene editing method suitable for chondrocyte populations from different cartilaginous sources.

**Methods:**

We screened electroporation and lipid nanoparticles for ribonucleoprotein (RNP) delivery in primary polydactyly chondrocytes, and optimized RNP reagents assembly. We knocked out RELA (also known as p65), a subunit of the nuclear factor kappa B (NF-κB), in polydactyly chondrocytes and further characterized knockout (KO) cells with RT-qPCR and Western Blot. We tested *RELA* KO in chondrocytes from diverse cartilaginous sources and characterized their phenotype with RT-qPCR. We examined the chondrogenic potential of wild-type (WT) and KO cell pellets in presence and absence of interleukin-1β (IL-1β).

**Results:**

We established electroporation as the optimal transfection technique for chondrocytes enhancing transfection and editing efficiency, while preserving high cell viability. We knocked out RELA with an unprecedented efficiency of ~90%, confirming lower inflammatory pathways activation upon IL-1β stimulation compared to unedited cells. Our protocol could be easily transferred to primary human chondrocytes harvested from osteoarthritis (OA) patients, human FE002 chondroprogenitor cells, bovine chondrocytes, and a human chondrocyte cell line, achieving comparable mean *RELA* KO editing levels using the same protocol. All KO pellets from primary human chondrocytes retained chondrogenic ability equivalent to WT cells, and additionally displayed enhanced matrix retention under inflamed conditions.

**Conclusions:**

We showcased the applicability of our bulk gene editing method to develop effective autologous and allogeneic off-the-shelf gene therapies strategies and to enable functional genetics studies in human chondrocytes to unravel molecular mechanisms of cartilage diseases.

**Supplementary Information:**

The online version contains supplementary material available at 10.1186/s13075-024-03294-w.

## Introduction

The clustered regularly interspaced short palindromic repeats and CRISPR-associated protein 9 (CRISPR-Cas9) system has become an effective genome engineering tool to modify DNA in a targeted manner, allowing modeling of molecular pathways towards a desired phenotype [1]. The original bacterial system consisting of a CRISPR-RNA (crRNA) paired with a trans-activating crRNA (tracrRNA) has been engineered into one single RNA transcript to yield a single guide RNA chimera (sgRNA). Such system allows a design for any genomic target by changing only the 20-nucleotide sgRNA spacer sequence, provided that a 5’-NGG-3’ protospacer adjacent motif (PAM) is contiguous to the targeted sequence [1]. Cas9 generates a double-strand break in the DNA, which is subsequently repaired by cells using alternative DNA repair pathways [2]. In particular, the non-homologous end joining machinery is error-prone and leads to stochastic nucleotide insertions and/or deletions (indels). Upon targeting a gene open reading frame (ORF), indels generation will inevitably cause a frameshift, thereby resulting in gene KO [3]. To date, the CRISPR-Cas9 technology has been employed in a multitude of cell lines for genome-wide screenings to identify and understand molecular pathways involved in biological processes [4].

However, gene editing in primary cells remains challenging due to difficulties in efficiently transfecting these cells. The reasons for this phenomenon are still elusive, but differences in transfection rates, cytokine production upon nucleic acid delivery, and DNA repair modalities may be contributors [2]. Traditional cell lines gene editing workflow involves selection of the edited cells through clonal expansion [4]. While this ensures that all cells harbor the same genetic modification, this approach is not always feasible for primary cells, as they do not possess infinite proliferation abilities and cannot be expanded as single cells [3, 5].

Such technical challenges are characteristic of primary human chondrocytes, as they dedifferentiate into a fibroblast-like state when cultivated in monolayer, have limited proliferation abilities and are resistant to nucleic acid delivery [6, 7]. Currently, investigated gene therapies for OA and rheumatoid arthritis (RA) involve recombinant adeno-associated viral vectors (rAAV), intraarticular delivery and retroviral vectors ex vivo gene delivery [8]. Examples include delivery of rAAV encoding chondrocytes transcription factors or growth factors using hydrogels or polymers [9, 10], or injection of rAAV harboring anti-inflammatory mediators [11]. In addition, ex vivo gene delivery in autologous synovial fibroblasts and allogeneic chondrocytes has been the focus of multiple RA clinical trials [8, 12, 13]. While undoubtedly efficient, these approaches carry significant limitations, including rAAV packaging [14], immunogenicity [15], pre-existing synovial antibodies targeting AAV capsid proteins [9], and the risk of genomic integration, albeit low [16]. In vitro functional genetics studies successfully mapped single-nucleotide-polymorphisms (SNPs) involved in multiple cartilage diseases [17–20] and investigated targeted gene KO and related implications on OA [21]. However, they all required viral-based CRISPR-Cas9 editing and either the generation and subsequent redifferentiation of induced pluripotent stem cells (iPSCs), or the use of cell lines. These cell sources have several limitations since they do not fully resemble the native molecular properties of human cartilaginous tissue [22].

Delivery of a Cas9-RNP complex is a safer strategy wherein the Cas9 is complexed with the sgRNA in vitro and subsequently delivered to the cells through physical methods (e.g., electroporation) or nanomaterial-based methods (e.g., lipid nanoparticles (LNPs)) [23]. The RNP is cleared from the cells rapidly, thus reducing off-target effects and immune responses [24]. Electroporation uses an electrical pulse to induce transient pores in the cellular membrane and facilitate biomacromolecule transfection [25]. Conversely, cationic liposomes encapsulate hydrophilic macromolecules within their amphiphilic bilayer, allowing cellular delivery via endocytosis or phagocytosis [26]. RNP-based bulk gene editing in primary chondrocytes has been performed to KO matrix metallopeptidase 13 (*MMP13*), with editing efficiency ranging from 63 to 74% [27]. Higher editing percentages (~ 90–98%) were achieved only upon delivery of two sgRNAs and a double transfection to target microRNA 140 [28].

**Fig. 1:**
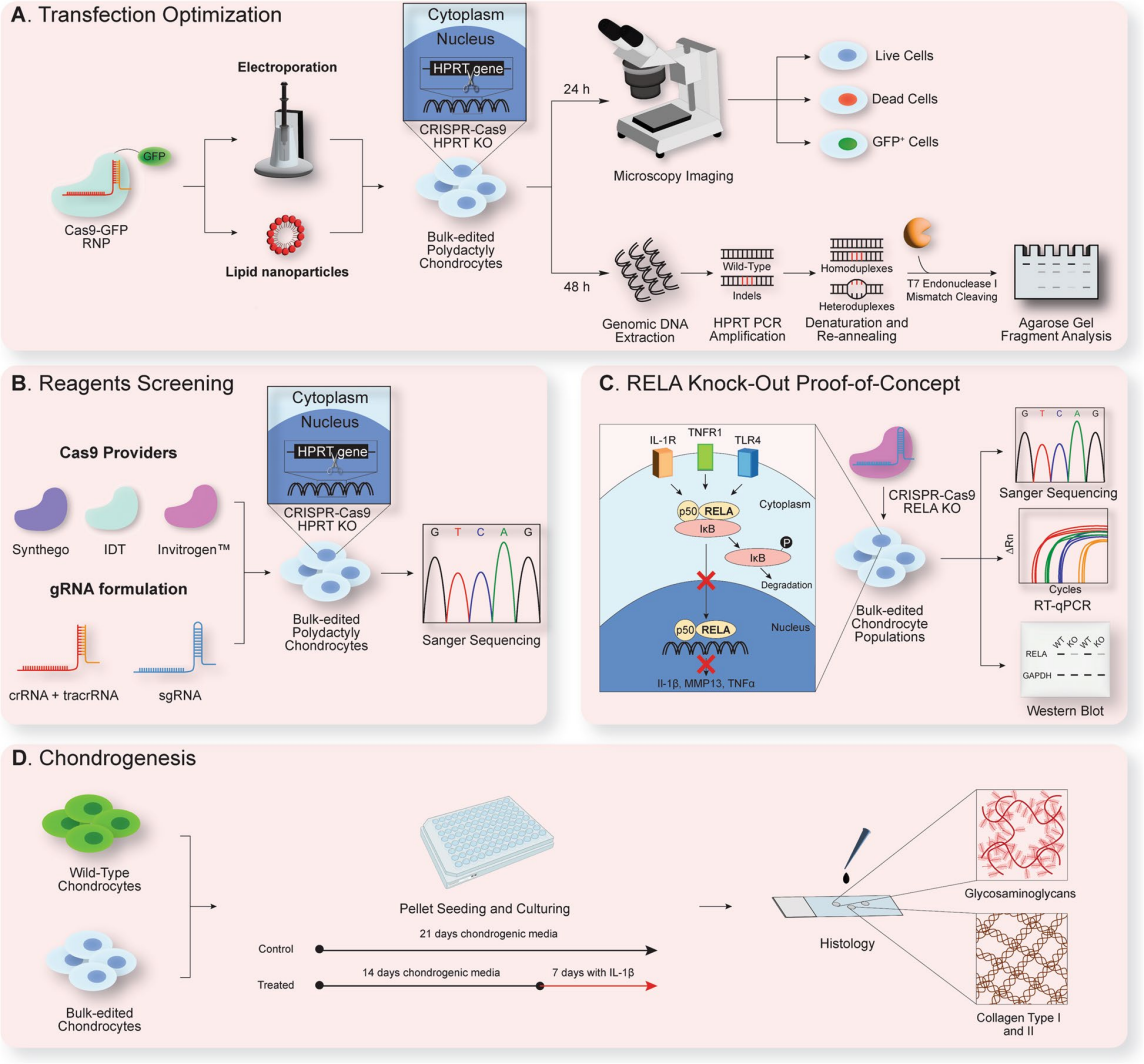
Overview of the experimental workflow outlined in this study. **A** Transfection optimization in primary polydactyly chondrocytes was performed by delivering a green fluorescent protein (GFP)-labelled Cas9-RNP targeting the housekeeping hypoxanthine guanine phosphoribosyltransferase (*HPRT*) gene, comparing electroporation and lipid nanoparticle delivery. After 24 h, microscopy imaging was used to infer transfection-associated cytotoxicity and efficiency, by counting live, dead and GFP^+^ cells, respectively. At 48 h post-delivery, DNA was extracted and the *HPRT* locus was amplified by polymerase chain reaction (PCR). Upon denaturation and re-annealing, mismatched heteroduplexes arising from base-pairing of WT and edited alleles were cut using the T7 endonuclease 1 (T7E1) enzyme. The generated fragments were run on a 1.2% agarose gel and editing efficiency was calculated. **B** Reagents optimization was carried out by testing three different Cas9 enzymes and two gRNA formulations, respectively. Editing efficiency at the *HPRT* locus in polydactyly chondrocytes was validated with Sanger sequencing. **C** Using our optimized parameters and reagents, we generated bulk-edited chondrocyte populations harboring a *RELA* KO. RELA is an essential component of the NF-κB complex, which regulates the activation of several pro-inflammatory cellular pathways (e.g., interleukin-1 beta (*IL-1*β), *MMP13* and tumor necrosis factor alpha (*TNFα*)). Besides calculating editing efficiency, we further characterized KO polydactyly chondrocytes with quantitative reverse transcription PCR (RT-qPCR) and Western Blot. Applicability and reproducibility of our KO gene editing technique was further tested in OA-patient-derived chondrocytes, FE002 primary chondroprogenitors, a human chondrocyte cell line and bovine chondrocytes, with *RELA* KO efficiency calculated using Sanger sequencing and phenotype validated via qPCR. **D** WT and KO pellets for all cell types were cultured for 3 weeks in chondrogenic media, with and without the addition of 10 ng/ml of IL-1β throughout the last week of culture. Extracellular matrix (ECM) deposition was assessed using histological staining for detecting glycosaminoglycans (GAGs) and immunostaining to verify the deposition of collagen type I and type II fibers

Recently, our group reported the potential of CRISPR-Cas9 gene editing for cartilage tissue engineering applications achieving high editing efficiency in targeting the transforming growth factor-β-activated kinase 1 (*TAK1*) gene [29]. Hence, we set out to establish a single-step, efficient CRISPR-Cas9 KO gene editing method in primary human chondrocytes that simultaneously maximizes cell viability, transfection and editing efficiency (Fig. 1). We optimized our workflow in polydactyly chondrocytes first, due to their enhanced proliferation and chondrogenic ability, ideal requisites for a thorough screening of transfection conditions and editing reagents [13, 30, 31]. We then investigated delivery of a Cas9-RNP by comparing LNPs and electroporation, respectively. Subsequently, diverse Cas9 enzymes and guide RNA (gRNA) formulations were screened to optimize the RNP assembly. We next proceeded with a RELA KO proof-of-concept. RELA is a subunit of the NF-κB complex [32], which triggers multiple cellular pro-inflammatory pathways that ultimately lead to articular joint degradation and OA onset [33]. RELA was chosen as our selected target as it is a well-characterized protein within the context of OA pathogenesis and inflammation [33]. Moreover, there are multiple commercially available control KO kits targeting this gene, to guarantee reproducibility of editing efficiency. We characterized the KO cell pool by Western blot and quantitative reverse transcription PCR (RT-qPCR). Next, we tested the reproducibility of our RELA KO in multiple alternative cell sources (i.e., clinical grade FE002 primary chondroprogenitors, the C28/I2 chondrocyte cell line, and OA-patient or bovine chondrocytes) and characterized their phenotype with RT-qPCR. Lastly, we examined retention of chondrogenic potential in KO cell pellets with respect to their WT counterpart and examined matrix production and retention under inflamed conditions. We envision such widely applicable technique will allow the development of allogeneic and autologous therapeutical gene editing strategies and functional genetics studies in chondrocytes deriving from multiple sources.

## Material and methods

### Cell culture 

Primary infant chondrocytes were collected from corrective surgeries of polydactyly patients aged 8–27 months after informed parents’ consent, while human osteoarthritic chondrocytes were procured during joint replacement surgeries from adult patients after informed consent (Kantonale Ethikkommission Zürich, license number PB_2017-00510). Cell isolation was carried out as described previously [30].

The C28/I2 human chondrocyte line has been used in the evaluation of chondrocyte-mediated changes associated with arthritis [34]. It was donated, courtesy of Professor Mary B. Goldring; Hospital for Special Surgery/Weill Medical College of Cornell University, New York, New York, USA.

The FE002 primary chondroprogenitor cell source was established from the FE002 organ donation, as approved by the Vaud Cantonal Ethics Committee (University Hospital of Lausanne–CHUV, Ethics Committee Protocol #62/07: “Development of fetal cell banks for tissue engineering”, August 2007). The FE002 donation was registered under a federal cell transplantation program (i.e., Swiss progenitor cell transplantation program). The epiphyseal cartilage biopsy was collected and processed multimodally for primary cell type establishment and clinical grade cell banking, as succinctly described previously [35].

Bovine chondrocytes were harvested from knees of 6-month-old calves, obtained from the local slaughterhouse, as previously described [36].

All cells were cultured in expansion medium composed of DMEM-GlutaMAX (Gibco 61965059, Waltham, Massachusetts, USA) supplemented with 10% v/v fetal bovine serum (Gibco 10270106, Waltham, Massachusetts, USA), 5 ng/ml of FGF2 (Peprotech 100-18B, Cranbury, New Jersey, USA) and 10 µg/ml of gentamicin (Gibco 15710049, Waltham, Massachusetts, USA). Cultures were grown in a humidified 5% CO_2_ atmosphere at 37°C. To perform gene editing experiments, primary cells were thawed at passage 1 and were left to recover for 4 days.

### Pellet culture

To induce pellet formation, 250,000 cells were resuspended in chondrogenic medium (DMEM-GlutaMAX [Gibco 61965059, Waltham, Massachusetts, USA] supplemented with 1% ITS [Gibco 41400045, Waltham, Massachusetts, USA], 40 µg/ml 1L-proline [Sigma-Aldrich P5607-25g, Burlington, Massachusetts, USA], 50 µg/ml ascorbic acid [TCI A2521-5G, Portland, Oregon, USA], 10 µg/ml gentamicin [Gibco 15710049, Waltham, Massachusetts, USA], and 10 ng/ml TGFβ3[Peprotech AF-100-36E-50ug, Cranbury, New Jersey, USA]) and centrifuged in a low attachment conical bottom 96-well-plate (Thermo Scientific 249570, Waltham, Massachusetts, USA) at 250 × g for 5 min. Pellets were cultured for 21 days, with media changes every second day. Pellets were stimulated with 10 ng/ml IL-1β (Peprotech 200-01B, Cranbury, New Jersey, USA) during the last 7 days of pellet culture. Untreated WT and KO pellets were used as a control.

### Gene editing reagents

Cas9-GFP was ordered from IDT (Alt-R™ S.p. Cas9-GFP V3 10008100, Coralville, Iowa, USA). For the reagents screening, we compared Cas9 from IDT (Alt-R™ S.p. Cas9 Nuclease V3 1081058, Coralville, Iowa, USA), Synthego (SpCas9 2NLS Nuclease, Redwood City, California, USA) and Invitrogen™ (TrueCut™ Cas9 Protein v2 A36498, Waltham, Massachusetts, USA).

HPRT gRNA was derived from the IDT KO control kit (IDT 1072554, Coralville, Iowa, USA). The sequence cannot be disclosed due to proprietary information. crRNA and tracrRNA were resuspended at 200 µM in the provided buffer, mixed in equal amounts and incubated at 95°C for 5 min. The reaction was diluted 1:6 with water to reach a final concentration of 17 µM. HPRT sgRNAs were ordered from IDT and Synthego, resuspended in the respective buffer to reach 100 µM, then diluted to a 30 µM working solution. RELA sgRNAs #1, #2, #4 were designed using the IDT design webtool (Custom Alt-R™ CRISPR-Cas9 guide RNA | IDT (idtdna.com)). sgRNA #5 was designed with the ChopChop webtool (CHOPCHOP (uib.no)), while sgRNA #3 was derived from the CRISPRevolution Control Kit from Synthego. All RELA sgRNAs were ordered from Synthego. RELA sgRNAs sequences, on-target, and off-target scores are listed in Supplementary Table 1.

### Transfections

Electroporation was performed using a Neon™ Transfection System 10 μL Kit and a Neon™ Transfection System (Invitrogen™ MPK1025, Waltham, Massachusetts, USA) following manufacturer’s protocol. Briefly, 7.5 pmol of Cas9 were incubated with 15 pmol of cr:tracrRNA/sgRNAs in 3.85 µl of buffer R for 10 min. 120,000 cells per reaction (resuspended in 5 μl Buffer R) were mixed with 5 μl of RNP and electroporated. Cells were then transferred into a T25 flask with 5 ml of pre-warmed expansion medium. 30 pmol of sgRNA were used for the 1:4 condition and 45 pmol for the 1:6 condition.

For LNPs, transfections were performed following manufacturers’ protocols.

For Lipofectamine™ 3000 (Invitrogen™ L3000001, Waltham, Massachusetts, USA), 10^5^ cells per reaction were seeded per well in 24-well plates the day before the experiments in 1 ml DMEM-GlutaMAX without supplements. 7.5 pmol of Cas9-GFP were mixed with 15 pmol of gRNA and 2 µl of P3000 reagent in 25 µl of OptiMEM (Gibco 31985062, Waltham, Massachusetts, USA) and incubated for 5 min. Then, 0.75 µl of Lipofectamine™ 3000 reagent were mixed with 25 µl of OptiMEM and added to the other mixture. 50 µl were added to each well of the plate.

For Lipofectamine™ RNAiMAX (Invitrogen™ 13778075, Waltham, Massachusetts, USA), 7.5 pmol of Cas9-GFP were mixed with 15 pmol of gRNA in 23.85 µl of OptiMEM per reaction and incubated for 5 min. Cells were trypsinized (Gibco 252000056, Waltham, Massachusetts, USA) and resuspended at a density of 10^6^ cells/ml in OptiMEM. Then, 1.2 µl of RNAiMAX reagent were added to 25 µl of the RNP mix and subsequently added to 100 µl of the cells, before being plated in a 24-well plate with 1 ml of pre-warmed expansion media.

For FuGENE® (Promega E2311, Madison, Wisconsin, USA), 10^5^ cells per reaction were seeded per well in 24-well plates the day before the experiment in 1 ml DMEM-GlutaMAX without supplements. 15 µl of FuGENE® were added to 45 µl of OptiMEM. Then, 7.5 pmol of Cas9-GFP were incubated with 15 pmol of gRNA for 5 min and were added to the FuGENE® mix. 50 µl of the mixture were added per well.

### Transfection efficiency and viability assessment

Propidium iodide (PI, Fluka 81845-25MG, Burlington, Massachusetts, USA) was added to polydactyly chondrocytes 24 h after transfections. Wells were imaged with three pictures per well using a Zeiss Axio Observer Z1 microscope using a 10X objective (Zeiss, Jena, Germany). GFP + cells were manually counted using Fiji software [37].

OA chondrocytes, C28/I2 cell line, chondroprogenitors and bovine chondrocytes were trypsinized, centrifuged 5 min at 500 × g and resuspended in 1 ml expansion media. 18 μl of the cells were transferred in a separate vial, and 2 μl of Acridine Orange live and PI dead stain solution (Logos Biosystem F23001, Annandale, Virginia, USA) were added to the cells. 10 μl of the mix were loaded onto a LUNA-FX7™ Automated Cell Counter (Logos Biosystems L70001, Annandale, Virginia, USA) to measure cell viability.

### T7E1 assay

To extract DNA, cells were resuspended in PCR buffer (Promega M7911, Madison, Wisconsin, USA) with 100 µg/ml of Proteinase K (Sigma-Aldrich 3115828001, Burlington, Massachusetts, USA), incubated at 55°C for 1 h and at 80°C for 15 min to inactivate the enzyme. DNA was amplified by Q5 polymerase (New England BioLabs E0555L, Ipswich, Massachusetts, USA). HPRT primers are listed in Supplementary Table 2. T7E1 assay was performed following manufacturer’s protocol (IDT 1075931, Coralville, Iowa, USA). PCR amplicons were mixed with T7E1 Reaction Buffer and water, heated in a PCR thermocycler at 95°C for 10 min, cooled first to 85°C (-2°C/sec), and subsequently to 25°C (-0.3°C/sec). Resulting duplexes were incubated with 2 µl of T7E1 enzyme (1 U/µl). Fragments were run on a 1.2% agarose gel (Promega V3121-500g, Madison, Wisconsin, USA) and editing efficiency was quantified using FIJI software [37].

### Histological analysis

After 21 days of chondrogenic stimulation, cell pellets were harvested and fixed with 4% paraformaldehyde solution (Merck 1004969011, Rahway, New Jersey, USA). Samples were dehydrated with serial passages in ethanol and processed with a LogosJ tissue processor (Milestone Medical SRL, Bergamo, Italy), then embedded in paraffin and cut into 5 μm thin slices. Samples were rehydrated with xylene and a decreasing ethanol cascade before staining. To detect glycosaminoglycans, samples were stained with Safranin O staining (Sigma-Aldrich S8884-25g, Burlington, Massachusetts, USA) according to standard protocols. Briefly, samples were stained in Weigert’s Hematoxylin solution (Sigma-Aldrich, H7107-500ML and HT109-500ML, Burlington, Massachusetts, USA) for 5 min, washed 3 times in dH2O, differentiated in 1% acid-alcohol (5 mL HCL 37% in 500 mL 70% EtOH) for 2 s, and washed 3 times in dH2O. Samples were stained in 0.02% Fast Green solution (Sigma-Aldrich F7252-5g, Burlington, Massachusetts, USA), washed in acetic acid for 30 s and stained in Safranin O solution for 30 min. Slides were rinsed in 95% EtOH and coverslips were mounted. Collagen type I and II immunostainings were performed after antigen retrieval for 30 min at 37°C with 1200 U/ml hyaluronidase (Sigma-Aldrich H3506-1G, Burlington, Massachusetts, USA) and 1 h blocking with 5% BSA in PBS (ITW reagents, A1391,0100, Monza, Italy). Primary antibody was added overnight at 4°C (mouse anti collagen I, Abcam, ab6308, 1:1000, working concentration 1.3 μg/ml; mouse anti collagen II, DSHB, II-II6B3, 1:20, working concentration 3.55 μg/ml, Cambridge, UK) in 1% BSA solution. Secondary horseradish peroxidase (HRP)-conjugated goat anti-mouse (Abcam, ab6789, Cambridge, UK) were diluted 1:1000 (working concentration 2 μg/ml) in 1% BSA solution and incubated for 1 h at RT. Development of staining was performed with a DAB substrate kit (Abcam, Cambridge, UK). Nuclei were counterstained with Weigert’s hematoxylin (Sigma-Aldrich HT109-500ML, HT107-500ML, Burlington, Massachusetts, USA). Samples were subsequently rinsed in 1% acid alcohol solution and treated with bluing agent (0.5 g sodium bicarbonate in 500 mL dH_2_O). Imaging was performed using the digital slide scanner Panoramic 250 Flash III by 3DHistech (Budapest, Hungary). Infant articular cartilage was obtained from corrective surgeries of polydactyly patients aged 8–27 months after informed parents’ consent, and used as a histological control (Kantonale Ethikkommission Zürich, license number PB_2017-00510). Briefly, cartilage was cut in thin sections with a scalpel, fixed with 4% paraformaldehyde solution and incubated in decalcifying solution (10% NH_4_-EDTA) for 7 days on a bench top lab roller (IKA 0004011000, Staufen, Germany). Subsequently, samples were embedded and cut as described above. Mouse IgG isotype controls were used as a negative control for both collagen type I and II antibodies (Invitrogen™ 31903, Waltham, Massachusetts, USA). Image quantification was carried out with Fiji [37]. In details, three 40X ROI screenshots per pellet were acquired. The color deconvolution function was used to separate the Safranin O/Collagen staining from the background blue and white colors. We measured the resulting pixel signal intensity and converted it into a logarithm, setting the WT untreated conditions as our control for each cell population. The intensities of the other experimental conditions (i.e., WT treated, KO treated and KO untreated) were then normalized to the WT control and all the values were multiplied by 100.

### Sanger sequencing

DNA was extracted and amplified as described above. Amplicons were run on an agarose gel, purified through a Wizard PCR and gel purification system (Promega A9281, Madison, Wisconsin, USA) and sent for sequencing at Microsynth AG (Balgach, Switzerland). Analysis of indels was performed through TIDE [38] (shinyapps.datacurators.nl/tide/). RELA genotyping and sequencing primers are listed in Supplementary Table 2. RELA predicted off-targets were identified with the IDT CRISPR-Cas9 guide RNA design checker (Custom Alt-R™ CRISPR-Cas9 guide RNA | IDT (idtdna.com)). Off-target primer sequences, number of mismatches, PAM, predicted IDT score and genomic locations are displayed in Supplementary Table 3. All genomic PCR primers designed in this study were ordered through Microsynth AG (Balgach, Switzerland).

### RNA extraction and RT-qPCR

WT and KO cells were plated in triplicate for every condition and for every donor in a 24-well plate (*n* = 9). Cells proliferated for two days and were cultivated in serum deprivation for 24 h. IL-1β (Peprotech 200-01B, Cranbury, New Jersey, USA) was added at a concentration of 10 ng/ml and RNA was collected after 16 h with NucleoZOL (Macherey Nagel 740404.200, Oensingen, Switzerland) following manufacturer’s protocol. 1 µg of RNA was retrotranscribed with GoScript Reverse Transcriptase kit (Promega A5003, Madison, Wisconsin, USA). cDNA was diluted 1:5 with water. RT-qPCR was performed with GoTaq qPCR Master Mix (Promega A6002, Madison, Wisconsin, USA). Reactions were run on a QuantStudio 3 96-well 0.1 ml Block Real-Time PCR System (Applied Biosystems™, Waltham, Massachusetts, USA). Samples were analyzed using the amplification and melt curves. Ct values were normalized to GAPDH, and genes expression was represented as fold changes over control condition. RT-qPCR primers are listed in Supplementary Table 4. All RT-qPCR primers designed in this study were ordered through Microsynth AG (Balgach, Switzerland).

### Western blot

WT and KO cells were plated in a 6-well plate (HUBERLAB 7.657 160, Aesch, Switzerland) and allowed to proliferate for two days prior to IL-1β (Peprotech 200-01B, Cranbury, New Jersey, USA) stimulation for 30 min at 10 ng/ml, as this was shown to be the optimal incubation time to allow NF-κB nuclear translocation [39]. Cells were lysed in RIPA buffer supplemented with protease inhibitors (Sigma-Aldrich P1860-1ML, Burlington, Massachusetts, USA) and then centrifuged for 10 min at 12,000 × g. Total protein contents were calculated using Pierce 660 nm Protein Assay (Thermo Scientific™, 22660, Waltham, Massachusetts, USA). 16 µg of proteins were mixed with NuPAGE™ Sample Reducing Agent (Invitrogen™ NP0004, Waltham, Massachusetts, USA) and NuPAGE™ LDS Sample Buffer (Invitrogen™ NP0007, Waltham, Massachusetts, USA) and denatured for 10 min at 80°C. Samples were run on a NuPAGE™ 4–12%, Bis–Tris, 1.0–1.5 mm, Mini Protein Gels (Thermo Scientific™ NP0321BOX, Waltham, Massachusetts, USA) and transferred onto a nitrocellulose membrane. Primary antibodies against GAPDH (Invitrogen™ PA1-987, Waltham, Massachusetts, USA, used 1:10,000, working concentration 0.1 μg/ml) and RELA (Cell Signaling Technology 76778, Danvers, Massachusetts, USA, used 1:1000, working concentration 0.1 μg/ml) were incubated with the membrane overnight at 4°C. Membranes were washed with PBS supplemented with 0.1% Tween and incubated with a secondary HRP-conjugated goat anti-rabbit IgG antibody (Abcam 6721, Cambridge, UK, used 1:1000, working concentration 2 μg/ml). The HRP signal was detected with WesternBright ECL HRP substrate (Advansta K-12045-D20, San Jose, California, USA) and imaged with a FUSION FX6 EDGE Imaging System (Witec, Sursee, Switzerland). Quantification of band intensity was performed using FIJI software.

### Statistical analysis

All data are presented with individual data points on the graphs, bar plots with error bars representing mean values ± SD. Statistical analysis was performed using the statistical analysis software GraphPad Prism 9.2.0 (GraphPad Software, San Diego, CA, USA, www.graphpad.com). Detailed statistical tests performed for each experiment and exact numbers of replicates “n” are stated in figure legends. *p* values are indicated as follows: * < 0.05, ** < 0.01, *** < 0.001, **** < 0.0001. *p* values < 0.05 were considered significant.

## Results

### Electroporation leads to more effective Cas9-RNP uptake and editing efficiency in chondrocytes compared to lipid nanoparticles

**Fig. 2:**
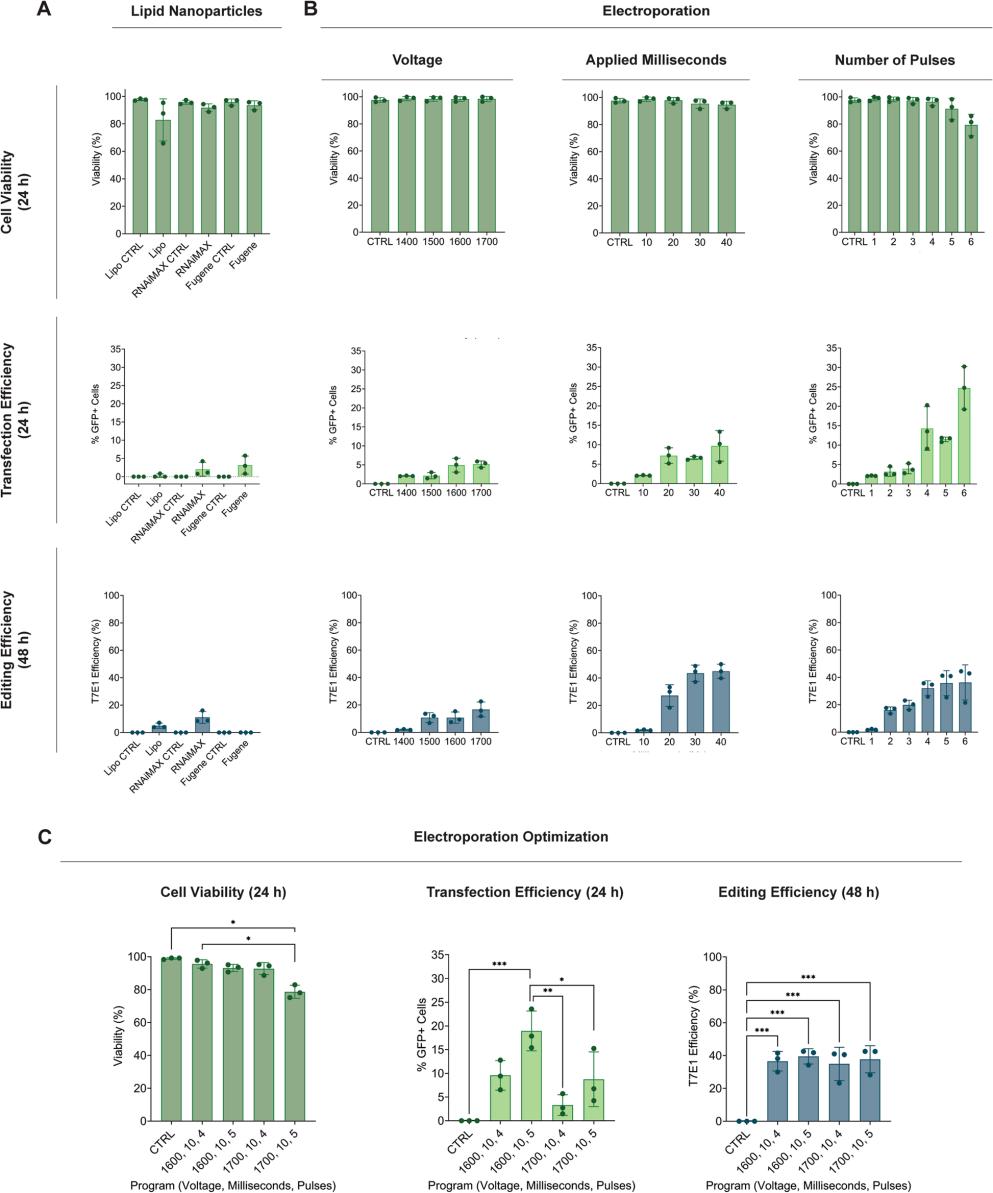
Electroporation leads to more effective transfection and *HPRT* editing efficiency compared to lipid nanoparticle delivery. Cell viability, transfection and editing efficiencies were calculated upon Cas9-RNP delivery with **A** the lipid nanoparticles Lipofectamine 3000™, Lipofectamine™ RNAiMAX and FuGENE® or the **B** Neon™ electroporation system, by testing different ranges of voltage, milliseconds or applied pulses, respectively. **C** Neon™ electroporation program optimization, calculating % of live cells, % of GFP^+^ cells and T7E1 efficiency. Non-transfected cells were used as viability controls, while cells transfected with an incomplete RNP, consisting of a Cas9-GFP without gRNA, were used as transfection and editing efficiency controls. Data are represented as mean ± standard deviation of 3 technical replicates from one donor (*n* = 3). Statistical significance was determined using one-way ANOVA with a Tukey’s multiple comparisons correction (* *p* < 0.05, ** *p* < 0.01, and *** *p* < 0.001)

We first investigated intracellular delivery of a Cas9-RNP targeting the *HPRT* gene in polydactyly chondrocytes, using a GFP-labelled Cas9 followed by microscopy imaging after 24 h comparing LNPs and electroporation, respectively. Total and dead cell counts were performed to assess transfection-related cytotoxicity, while GFP^+^ cell count was used to quantify transfection efficiency. Editing efficiency was calculated with a T7E1 assay 48 h after RNP delivery. LNPs led to good overall cell viability, but poor transfection efficiency. This finding was further corroborated by the T7E1 assay, which confirmed low editing levels for both tested Lipofectamines™ and the FuGENE® transfection reagent (Fig. 2A, Supplementary Figure 1A). Microscopy revealed that instead, LNPs gave rise to GFP^+^ aggregates (Supplementary Figure 1B).

As electroporation involves the generation of an electrical pulse, there are multiple parameters that can be optimized. For our Neon™ transfection apparatus these were voltage (V), applied milliseconds (ms) and the number of pulses, respectively. We screened a wide range for each of these parameters, while simultaneously keeping the other two constant. In details, when screening multiple voltages, 1 pulse of 10 ms was applied; when optimizing the time, 1400 V and 1 pulse was used and, finally, when testing different number of pulses, voltage and milliseconds were kept constant at 1400 V and 10 ms respectively (Supplementary Figure 2). We observed that increasing the number of pulses negatively affected cell survival, while increasing voltage, milliseconds and pulses led to increasingly higher transfection and editing efficiency (Fig. 2B, Supplementary Figure 2). A secondary optimization established that electroporation at 1600 V, for 5 pulses of 10 ms each led to the highest mean editing efficiency of 39.49 ± 4.63% and Cas9-GFP uptake (18.95 ± 4.19%), while simultaneously preserving a viability of 93.13 ± 2.22% (Fig. 2C). Unfortunately, the combination of 30 or 40 ms with the selected voltage and pulses values could not be tested, as well as the use of 30 and 40 ms with 4 or 5 pulses at the lowest 1400 V voltage, as it exceeded the maximum power capacity of the machine. Electroporation with 20 ms resulted in sparks formation throughout the transfection procedure, while transfection at 1700 V for 5 pulses of 10 ms each led to significantly lower cell viability (Fig. 2C, Supplementary Figure 3A). We excluded those conditions from further analyses. We did not observe major differences in the T7E1 assay across the different final conditions, however, we noticed the T7E1 efficiency to be on par with to the one we reported for 1400 V, 30 and 40 ms, 1 pulse in our initial screening (Fig. 2B). As the enzyme does not recognize and cleave 1 bp mismatches [38], we further confirmed *HPRT* editing with Sanger sequencing, which again confirmed that electroporation at 1600 V, 10 ms for 5 pulses lead to the highest mean editing efficiency of 67.03 ± 7.39% (Supplementary Figure 3B).

### Commercial CRISPR reagents formulation impact on genome editing optimization

**Fig. 3:**
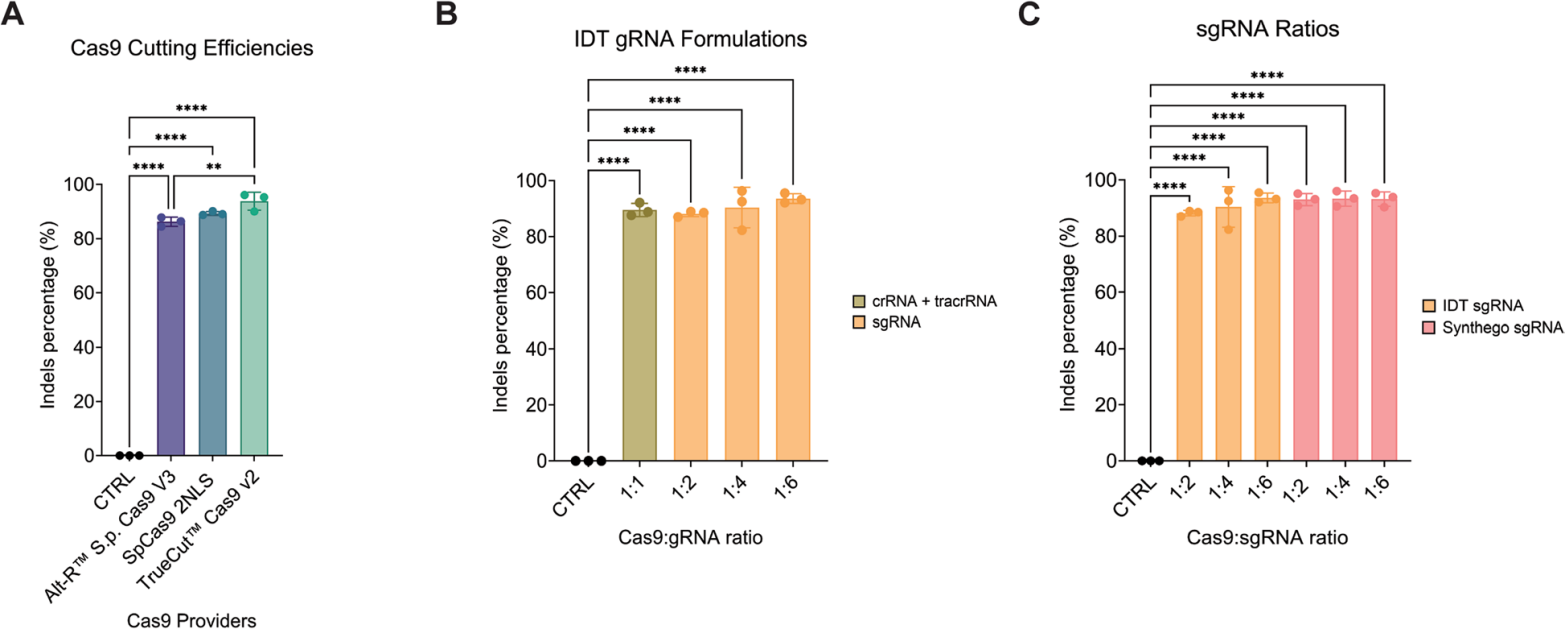
TrueCut™ Cas9 v2 and sgRNA provided by Synthego lead to high *HPRT* editing efficiency. Editing efficiency of the *HPRT* locus was calculated upon Cas9-RNP delivery testing while comparing **A** three different Cas9 manufacturers, **B** the gRNA synthesized as either a cr:tracrRNA complex or sgRNA at various Cas9:gRNA ratios and **C** two distinct sgRNA providers, testing multiple Cas9:sgRNA ratios. Data are represented as mean ± standard deviation of 3 technical replicates from one donor (*n* = 3). Statistical significance was determined using one-way ANOVA with a Tukey’s multiple comparisons correction (** *p* < 0.01, and **** *p* < 0.0001)

Next, we set out to evaluate the best transfection reagents to use for our RNP assembly. TrueCut™ v2 Cas9 achieved not only the highest *HPRT* mean editing efficiency (93.80 ± 3.32%), but its performance was higher than the Alt-R™ S. p. Cas9 V3 purchased from IDT (86.20 ± 1.71%), and the Synthego SpCas9 NLS nuclease (89.27 ± 0.74%) (Fig. 3A). We then compared the best gRNA formulation for RNP delivery. The bacterial crRNA precursor is base-paired with a scaffolding tracrRNA prior to Cas9 complexing [1]. Hence, it is possible to design the crRNA spacer sequence for the target of interest and separately order the scaffolding tracrRNA. A functional cr:tracrRNA duplex is then achieved upon complexing in a thermocycler. sgRNAs are more adaptable to high-throughput processing, as the tracrRNA is looped in an individual RNA molecule [1]. Moreover, sgRNAs can be chemically modified, resulting in RNA molecules with enhanced stability and thereby increased efficiency [2]. In addition, varying the Cas9:sgRNA ratio can improve editing efficiency, as an excess of sgRNA ensures Cas9 saturation, thereby decreasing the presence of free protein that can complex with intracellular competitors RNA molecules [40]. For this reason, we compared *HPRT* editing efficiency obtained with a cr:tracrRNA complex and with a sgRNA complexed with Cas9 at different ratios. While the cr:tracrRNA complex achieved high editing efficiency, using the sgRNA at a 1:6 ratio allowed us to reach even higher mean editing levels of 93.6 ± 1.74%, although this difference was not statistically significant (Fig. 3B). We finally examined *HPRT* editing efficiency comparing sgRNAs supplied by either IDT or Synthego. *HPRT* sgRNA ordered from Synthego and used at a 1:2 ratio already reached maximal editing efficiency of 93 ± 2.15%, comparably to the 1:6 IDT ratio (Fig. 3C). In conclusion, we selected the TrueCut™ v2 Cas9 enzyme and sgRNAs ordered from Synthego used at a 1:2 ratio for further experiments.

### Highly efficient, bulk RELA KO in a polydactyly chondrocytes population reduces pro-inflammatory pathways activation upon IL-1β stimulation

**Fig. 4:**
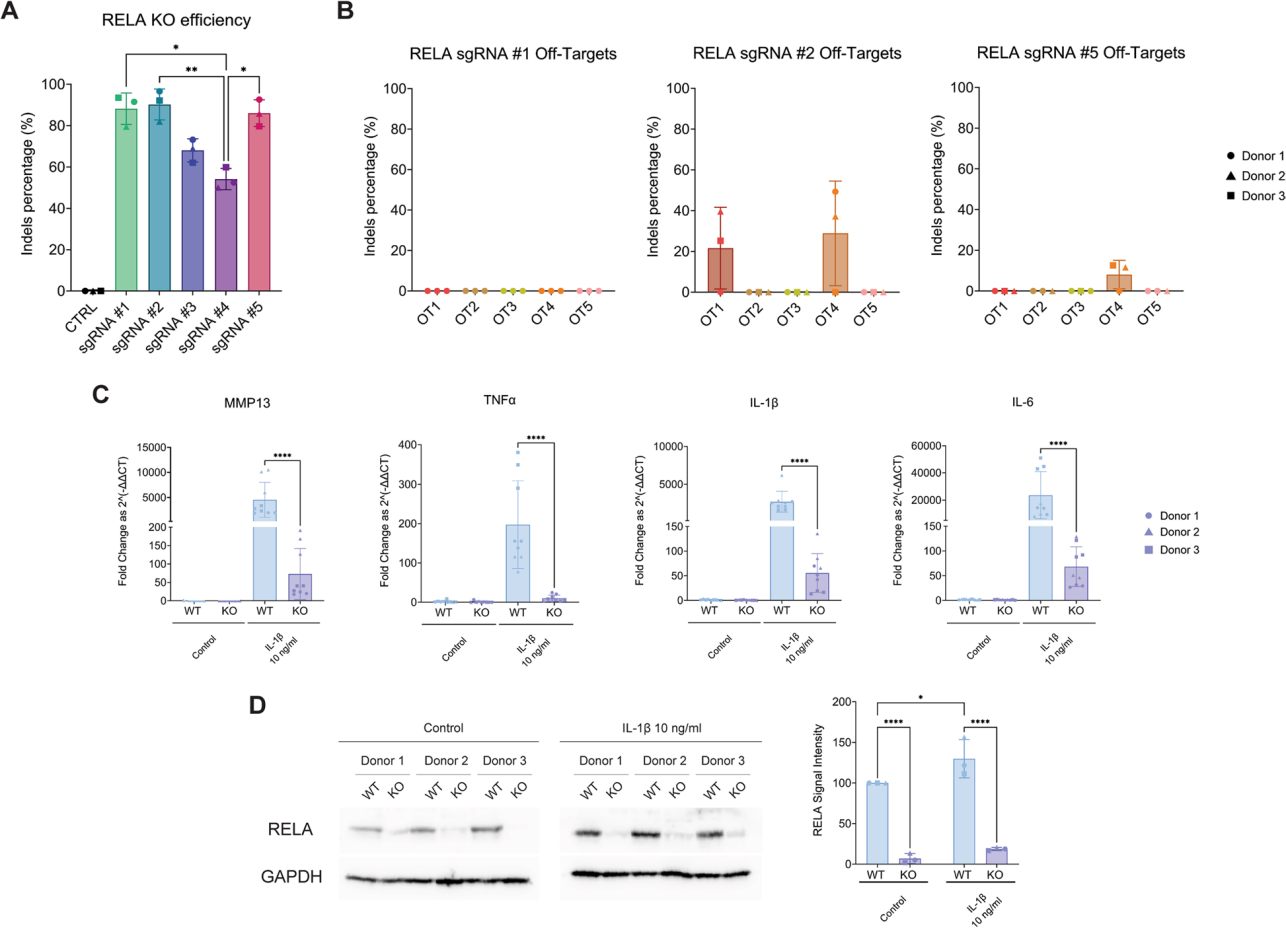
Sanger sequencing reveals highly efficient *RELA* KO in primary human polydactyly chondrocytes, and expression of NF-κB-dependent inflammatory pathways is lower in KO cells. **A** Editing efficiency of five different sgRNAs targeting the *RELA* gene at different locations. **B** Off-target effects of the best-performing sgRNAs #1, #2 and #5 at the top five in silico-predicted most probable off-target sites. Data are represented as mean ± standard deviation of 3 biological replicates from three donors (*n* = 3). Statistical significance was determined using a nonparametric one-way ANOVA (* *p* < 0.05, ** *p* < 0.01, and **** *p* < 0.0001). **C** RT-qPCR of selected inflammatory pathways acting downstream of NF-κB in WT and KO cells following stimulation with IL-1β for 16 h. **D** Western blot and quantification of RELA in WT and KO cells treated with IL-1β for 30 min compared to housekeeping glyceraldehyde-3-phosphate dehydrogenase (GAPDH) protein levels. RT-qPCR data are represented as mean ± standard deviation of 3 technical replicates from three biological donors (*n* = 9). Western Blot data are represented as mean ± standard deviation of 3 biological replicates from three donors (*n* = 3). Statistical significance was determined using a nonparametric Mann–Whitney t-test

We then investigated whether our optimized gene editing method could efficiently KO a target of our choice. We selected RELA, an essential constituent of the NF-κB complex. Physiologically, upon pro-inflammatory receptors activation, the cytoplasmic inhibitor of κB (IκB) protein is phosphorylated and degraded [41]. This event leads to the release of the NF-κB complex (i.e., a dimeric complex made up of RELA-p50), which in turn translocate in the nucleus and transcriptionally activates several pro-inflammatory genes [32]. We designed five sgRNAs targeting different exons of the *RELA* gene. sgRNAs #1, #2 and #5 led to comparably high editing efficiency with mean editing of 88.17 ± 7.57%, 90.23 ± 7.48% and 86.03 ± 6.45%, respectively (Fig. 4A). sgRNA #1 did not have any off-target activity, sgRNA #2 displayed off-target cutting activity in two out of the top five genomic off-target locations, while sgRNA #5 showed off-target effects in one DNA locus (Fig. 4B). Collectively, sgRNA #1 was selected as the best performing guide, and we further characterized the KO cells. NF-κB directs the activation of several pro-inflammatory pathways, including *MMP13*, *TNFα*, *IL-1*β and interleukin-6 (*IL-6*) [32, 33]. We hypothesized that upon RELA KO and IL-1β stimulation, NF-κB protein expression would be inhibited, resulting in reduced upregulation of downstream pathways. RT-qPCR showed that, as expected, all four pro-inflammatory pathways were upregulated in WT cells. The same trend was observed in KO cells, yet all pathways were activated several orders of magnitude lower (Fig. 4C). RELA protein levels in KO cells were investigated with Western Blot. Protein levels were found to be upregulated in both WT and KO cells upon addition of IL-1β. Nonetheless, KO cells displayed a much less potent upregulation (Fig. 4D, Supplementary Figures 4 and 5).

### RELA KO is reproducible with comparably high editing efficiency in a wide variety of chondrocytes populations

**Fig. 5:**
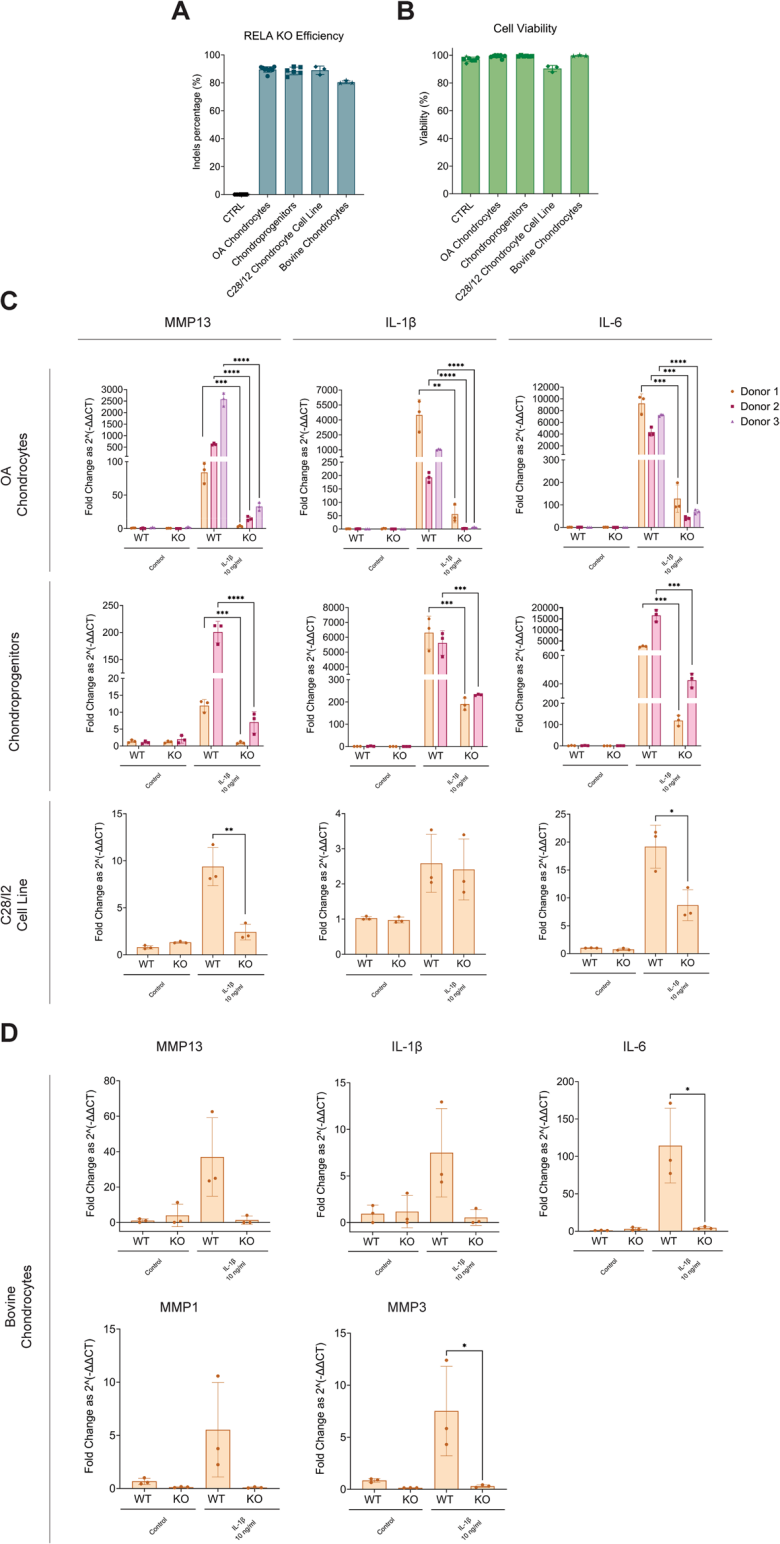
RELA KO is reproducible in a wide variety of primary chondrocyte populations and in a chondrocyte cell line with comparably high editing efficiency. (**A**) Editing efficiency and (**B**) cellular viability data of OA chondrocytes, human FE002 primary chondroprogenitors, the C28/I2 cell line and bovine chondrocytes edited with sgRNA #1 to induce a RELA KO. Cells mock-transfected with an incomplete RNP (i.e., Cas9 only, no sgRNA) were used as a control. RT-qPCR of selected inflammatory pathways acting downstream of NF-κB in (**C**) human and (**D**) bovine WT and KO cells following stimulation with IL-1β for 16 h. Data are represented as mean ± standard deviation of 3 technical replicates for bovine chondrocytes (*n* = 3) and the C28/I2 cell line (*n* = 3), of 3 technical replicates from two primary chondroprogenitor donors (*n* = 6), and of 3 technical replicates from three OA chondrocyte donors (*n* = 9). Statistical significance was determined using an unpaired t-test (∗*p *< 0.05, ∗∗*p* < 0.01, ∗∗∗*p* < 0.001, and ∗∗∗∗*p* < 0.0001)

Next, we investigated the applicability of our methods to induce an effective *RELA* KO using sgRNA #1 in chondrocytes derived from various tissue sources, i.e., OA-patient-derived chondrocytes, FE002 primary chondroprogenitor cells and the C28/I2 human chondrocyte cell line. We designed a sgRNA targeting the ortholog bovine *RELA* gene and tested its efficiency in bovine chondrocytes. sgRNA #1 was able to induce high *RELA* editing efficiency in all tested human chondrocytes, comparably to the editing in polydactyly chondrocytes. The sgRNA targeting bovine *RELA* gene also led to a high editing efficiency of 80.40 ± 1.04% (Fig. 5A). Upon cell harvesting and live/dead stain we found the percentage of viable cells to be high for all tested cell types (Fig. 5B). We subsequently investigated *MMP-13*, *IL-1β*, and *IL-6* pro-inflammatory pathways in WT and KO cells (Fig. 5C). In OA chondrocytes and chondroprogenitor cells, we observed a strong upregulation of all markers, which were in turn drastically less activated in KO cells. The C28/I2 cell line displayed similar, albeit milder, trends with respect to *MMP13* and *IL-6*, while *IL-1β* levels were unchanged between WT and KO groups, even upon IL-1β stimulation. Bovine chondrocytes showed trends for *MMP13* and *IL-1*β that were overall comparable to primary human cells, albeit not statistically significant (Fig. 5D). Only the *IL-6* pathway was found to be significantly less upregulated in RELA KO cells compared to WT. As bovine cells were reported to selectively activate matrix metalloproteinase-1 (MMP-1) and matrix metalloproteinase-3 (MMP-3) upon pro-inflammatory stimulation [42], we investigated those pathways. While RELA KO cells were found to upregulate *MMP1* much lower than WT cells, this difference was not statistically significant. We did however observe a statistically significant difference in the activation of the *MMP3* pathway between WT and KO bovine cells, with RELA KO chondrocytes showing a 15-fold lower upregulation. *TNFα* remained largely undetected in all samples and was thereby not included in our analysis.

### RELA KO chondrocyte pellets display strong retention of GAGs and Collagen type II in an inflamed environment

**Fig. 6:**
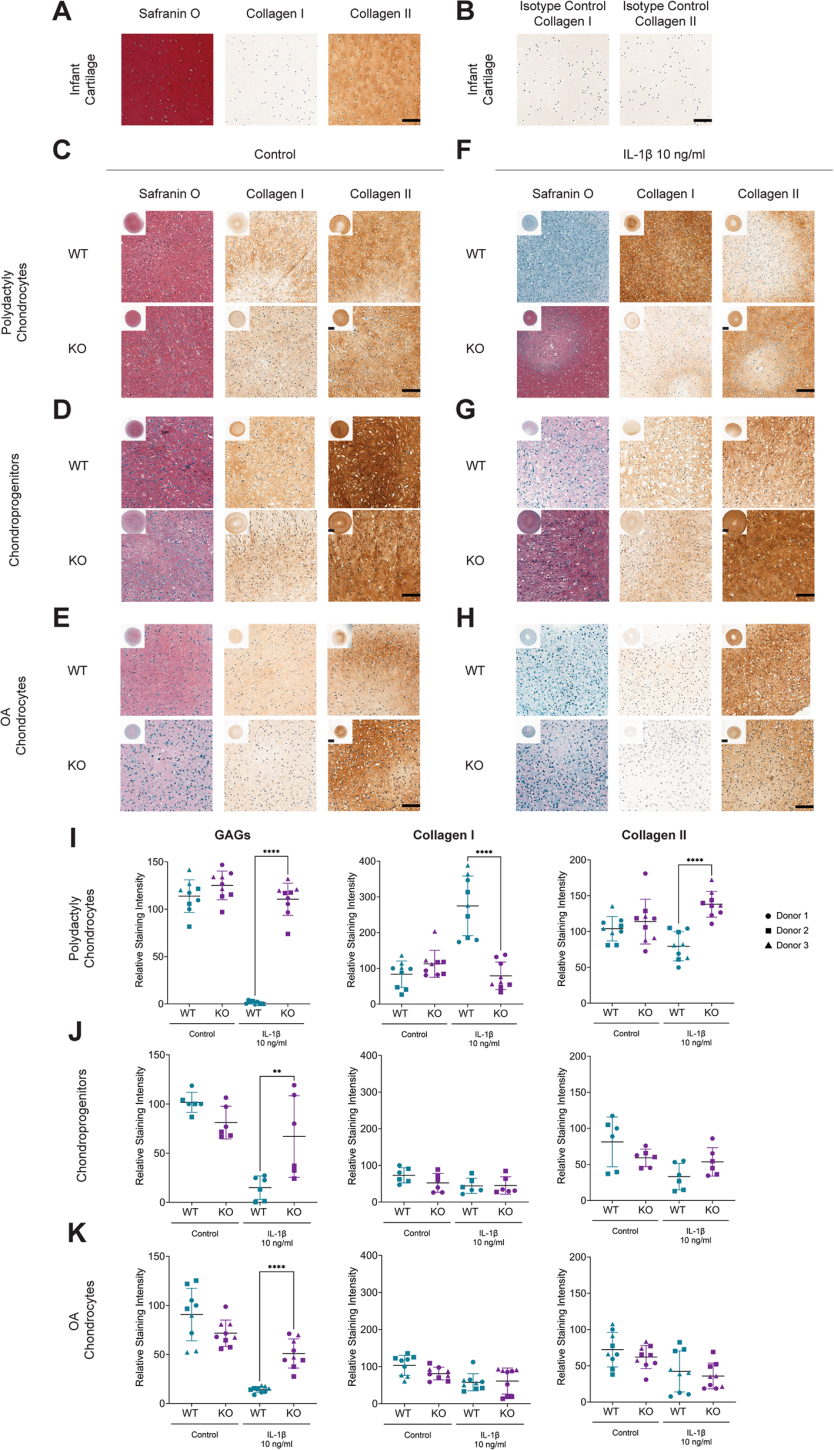
*RELA* KO chondrocyte pellets display ECM deposition compared to WT cells, and they are more resistant to IL-1β induced catabolism. Infant articular cartilage was used as a **A** positive and **B** negative histological control. Safranin O, collagen type I and II immunostaining of **C** polydactyly chondrocytes, **D** FE002 chondroprogenitor cells, and **E** OA chondrocyte pellets in an untreated condition after 21 days of culture in chondrogenic media. Safranin O, collagen type I and II immunostaining of **F** polydactyly chondrocytes, **G** FE002 chondroprogenitor cells, and **H** OA chondrocytes treated with 10 ng/ml of IL-1β in the last week of culture in chondrogenic media. **I**-**K** Quantification of the relative staining intensity. 20X pictures scale bar, 100 µm; low magnification 2X scale bar, 500 µm. One donor per cell type is showed as a representative example

Lastly, we set out to evaluate the chondrogenic potential of WT and KO cell pellets cultured for 21 days and to investigate whether RELA KO affected cartilage maturation. Control infant articular cartilage shows the characteristic abundance of GAGs and collagen type II, and low amount of collagen I (Fig. 6A), while isotype controls confirmed the specificity of our antibodies (Fig. 6B). All KO primary human cells showed comparable chondrogenic ability to their WT counterpart in 3 independent donors, (Fig. 6C-D, I-J, Supplementary Figure 6A-B). Chondrocytes from OA patients produced overall less GAGs, however the chondrogenic potential was maintained between WT and KO, as confirmed by images quantification (Fig. 6E,K, Supplementary Figure 6C). Simultaneously, we investigated matrix deposition of pellets exposed for the last week of culture to IL-1β, a key molecular player driving OA disease pathogenesis [43]. Strikingly, all primary human RELA KO cells were able to retain GAGs and collagen type II synthesis, while WT pellets showed significantly greater GAGs loss compared to KO pellets (Fig. 6F-K, Supplementary Figure 6D-F). While collagen type I and II levels remained unchanged (i.e., not statistically significant) in inflamed chondroprogenitors and OA chondrocytes, polydactyly chondrocytes displayed significantly reduced levels of collagen I and significantly higher amount of collagen II (Fig. 6I). WT and KO cell line pellets did not produce any GAGs and presented low levels of collagen type II in both untreated and inflamed condition (Supplementary Figure 7A-B). Finally, while chondrogenic potential of WT and KO bovine cells was unchanged in the control condition, IL-1β stimulation did not substantially affect GAGs and collagens production (Supplementary Figure 7C-D). Untreated pellets number of cells/mm^2^ was measured after 3 weeks of culture and was not significantly different between WT and KO (Supplementary Figure 7E).

## Discussion

The CRISPR-Cas9 system has revolutionized the field of biomedical research, enabling the development of gene therapy-based clinical strategies, and the in vitro modeling of cellular pathways to unravel genetic determinants of diseases. For cartilage regeneration, a multitude of CRISPR-Cas9-based studies have been reported [44]. However, they all involve the use of cellular models which do not recapitulate the physiological phenotype of human cartilage (e.g., iPSCs, cell lines [17–20]) and they employ viral vectors [9–11]. rAAVs require the use of specialized biosafety laboratory facilities and the associated safety concerns slow down their clinical application. In addition, reported cell lines are not capable of secreting well-matured ECM and, when they do, it is abnormally matured [45].

Here, we developed an RNP gene editing strategy to achieve strikingly high editing efficiency in bulk, generating a chondrocyte cell pool ready for downstream application, without the need for further enrichment. Our KO efficiency is considerably greater than in reported RNP analogous approaches [27, 28], and comparable to a recent work published by our group [29]. RNP delivery ensures transient editing, thereby reducing the risk of off-target effects and immunogenicity. Most importantly, our workflow is easily transferrable to chondrocytes from alternative sources, without requiring any parameter changes or adjustments.

We established electroporation as the gold standard for effective transfection of chondrocytes. While this is in line with previously published studies [7, 46], LNPs poor transfection and editing efficiency comes as a surprise, as FuGENE® and Lipofectamine™ were successfully employed as carriers for RNPs delivery [27, 28, 47]. However, suboptimal editing reported in previous approaches might specifically be a direct result of the poor transfection efficiency observed here. In particular, we noticed poor intracellular LNPs delivery, with GFP^+^ particles clustering on the cell membrane instead.

For electroporation, our best program at 1600 V for 10 ms and 5 pulses successfully balanced high transfection and *HPRT* cutting efficiency, while at the same time displaying extremely low cytotoxicity. Most notably, while the NEON electroporation apparatus is equipped with default protocols, we carried out the transfection optimization in-house by manually changing and testing different combinations of voltage, number of pulses, and time. In addition, previous literature available regarding transfection in chondrocytes using this specific device reports electroporation of plasmids, micro RNAs or messenger RNA (mRNA) [48–50], but not of CRISPR-Cas9 RNPs. Furthermore, our study showcased that the optimal bulk transfection efficiency was achieved by a combination of devices and reagents from at least 3 different commercial manufacturers, and an open-source CRISPR guide design algorithm.

The T7E1 assays did not detect major differences in the editing efficiency within the final four electroporation conditions, which comes out as an apparent discrepancy with the GFP^+^ cell count. We propose the following explanation. Microscopy can only return snapshots that represent Cas9 delivery at a specific time point. RNP editing kinetics were shown to reach a peak around 24 h, but previous editing events already took place, and more will still occur, plateauing at 48–72 h [24, 51]. For this reason, we coupled microscopy measurement with T7E1 assays to measure final-timepoint editing efficiency. However, the T7E1 enzyme cannot efficiently cut 1 bp mismatches, thus resulting in an underestimation [52]. Conversely, Sanger sequencing provides a much more precise readout.

We additionally screened gene editing reagents, to obtain the highest efficiency in RNP formulation. While differences were small and largely not statistically significant among Cas9 and sgRNA manufacturers and molar ratios, we argue that it is still crucial to choose the optimal conditions which lead to the highest mean editing efficiency. This is true especially for sgRNAs that show a low cutting efficiency or for knock-in experiments, where the PAM constraints often do not leave large margins for possible sgRNAs design. Finally, as bulk editing strategies inevitably result in the persistence of WT cells among the cell population, it is crucial to reliably obtain a KO cell pool with a distinct phenotype.

Sanger efficiency highlighted strikingly high *RELA* editing levels, which were further validated by the little levels of persisting protein. RT-qPCR confirmed low upregulation of NF-κB-associated downstream pro-inflammatory pathways, implying that we were indeed able to generate a chondrocyte population resistant to inflammatory stimuli. In principle, our method is easily adaptable to virtually any genomic target, with sgRNA design being the only parameter to be adjusted accordingly. As epigenetic status and chromatin accessibility might hinder efficiency [53] and cause differences in editing efficiency across different cell types [54], we recommend testing multiple sgRNAs. Off-target effects monitoring is also essential, especially for solutions destined for clinical translation. We advise using Sanger sequencing or even next generation sequencing, if possible, to obtain much deeper resolution.

We additionally showcased the applicability of our technique by replicating *RELA* KO in clinical grade FE002 primary chondroprogenitors, a human chondrocyte cell line, OA patient and bovine chondrocytes, achieving the same high editing efficiency. The phenotype obtained in OA chondrocytes and FE002 chondroprogenitors was overall comparable to the one observed in polydactyly chondrocytes, as confirmed by RT-qPCR, albeit we registered high donor-to-donor variability. This highlights how understanding patient-specific responses to external pro-inflammatory stimuli is crucial to better characterize the disease phenotype and potentially formulating personalized therapeutic strategies. Bovine chondrocytes overall followed the same trends observed in human cells, although we observed a much lower and not statistically significant activation of the IL-1β and MMP13 pathways, suggesting that bovine cells respond to IL-1β stimulus following distinct pathways compared to the human counterpart., In addition, while some groups reported higher activation of MMP-1 and MMP-3 in bovine cells [42], we could only observe a significant difference in the upregulation of *MMP3* between WT and RELA KO cells. We believe that the lower activation of the bovine pro-inflammatory pathways observed in this study might be due to the choice of IL-1β as the pro-inflammatory inducer. In fact, other groups used interleukin-1 alpha (IL-1α) to induce inflammation in bovine cells [55, 56]. The C28/I2 cell line did not upregulate any of the selected pathways to levels comparable to human primary cells. Strikingly, IL-1β levels remained completely unchanged in treated WT and KO cells. This does not come as a surprise, as the insensitivity of C28/I2 cells towards inflammatory stimuli has been reported in the literature, citing the fact that this cell line has not been generated from articular chondrocytes as the primary cause [57]. Currently, functional genetics studies in primary human cells to understand the molecular mechanisms underlying OA and other cartilage diseases, are needed. To date, iPSCs have been the cell line of choice to study genetic functional risk variants associated with OA [17–20]. However, iPSCs generation and redifferentiation carries significant limitations besides being technically laborious. Pluripotency induction often happens at low efficiency [58], and potential genetic abnormalities that could affect cell behavior upon redifferentiation must be ruled out [20]. Our approach effectively allows to study the impact of gene KO directly in primary chondrocytes, thereby resulting in a more accurate phenotype.

Finally, we demonstrated that KO pellets perfectly retain chondrogenic ability comparable to WT cells, establishing that our gene editing methods does not negatively impact chondrogenic potential of the cells. Most notably, human primary cell KO pellets were all able to retain GAGs and collagen II production capacities under inflamed conditions. This not only further strengthens the findings previously reported by our group [29], but radically expands the impact of bulk chondrocytes gene editing to potentially develop not only allogeneic therapies (with e.g., clinical grade FE002 chondroprogenitor cells and polydactyly chondrocytes), but also, for the first time, autologous gene therapy strategies with patients-derived OA chondrocytes. Autologous chondrocytes implantation (ACI) and matrix-induced ACI (MACI) are two cell-based therapies for cartilage regeneration that rely on the harvesting of a small biopsy, followed by autologous chondrocytes culturing ex vivo and subsequent re-implantation into the site of the lesion [59]. Despite being a commercially available product [60], ACI and MACI carry significant limitations. Chondrocytes dedifferentiate over the in vitro expansion, thereby leading to graft failure, and the two procedures are prone to donor site morbidity [61]. The combination of gene therapy with ACI and MACI has already been successfully explored with the use of viral vectors and plasmids [60]. Specifically, chondrocytes overexpressing TGF-β1 [62], FGF-2 [63] and IGF-1 [64] were found to enhance chondrogenesis in vivo, while synoviocytes expressing IL-1Ra and IL-10 [65] improved OA symptoms in rabbits. Conversely, here we present an important milestone towards bridging the gap between RNP ex vivo editing and cartilage regeneration, by generating clinically-grade genetically modified cells with enhanced regeneration potential. CRISPR-Cas9 RNP strategies are effectively already employed for a wide variety of other diseases, mainly for cancer immunotherapy [66], hematologic disorders [67] and immune system disorders [68]. While we presented here applications for autologous OA chondrocytes and allogeneic polydactyly chondrocytes and chondroprogenitors, this protocol can be easily tailored to other relevant cell types for cartilage regeneration and musculoskeletal diseases such as mesenchymal stem cells (MSCs). Furthermore, the device used in this study is available in a 100 µl version that allows transfection of 10^6^ cells at a time, and in a good manufacturing practice (GMP) version, allowing transposition of our streamlined gene editing process in GMP settings.

## Conclusions

In summary, we identified a novel gene editing strategy capable of inducing genes KO in a wide variety of human chondrocytes with unprecedented high editing efficiency. Overall, we envision our method to have a major impact on the cartilage research field, allowing deep understanding of molecular pathways driving cartilage diseases by e.g., studying SNPs leading to premature stop codons in genes ORF or exploring the impact of candidate genes KO on the overall chondrocytes phenotype (i.e., metabolism, proliferation and chondrogenic potential). Moreover, the combination of our editing protocol with tissue engineering techniques will have an impact in the regenerative medicine field, allowing the development of novel autologous and allogeneic gene therapy strategies.

### Supplementary Information

**Supplementary Material 1.**

## Data Availability

The data that support the findings of this study are openly available in ETH Research Collection at doi: 10.3929/ethz-b-000622382.

## Abbreviations

CRISPR-Cas9: Clustered Regularly Interspaced Short Palindromic Repeats and CRISPR-Associated Protein 9
RNP: Ribonucleoprotein
NF-Κb: Nuclear Factor Kappa B
KO: Knockout
RT-qPCR: Quantitative Reverse Transcription PCR
WT: Wild-Type
IL-1β: Interleukin-1β
OA: Osteoarthritis
crRNA: CRISPR-RNA
tracrRNA: Trans-activating crRNA
sgRNA: Single Guide RNA
PAM: Protospacer Adjacent Motif
indels: Insertions and/or Deletions
ORF: Open Reading Frame
RA: Rheumatoid Arthritis
rAAV: Recombinant Adeno-Associated Viral Vectors
SNPs: Single-Nucleotide-Polymorphisms
iPSCs: Induced Pluripotent Stem Cells
LNPs: Lipid Nanoparticles
MMP13: Matrix Metallopeptidase 13
TAK1: Transforming Growth Factor-β-Activated Kinase 1
GFP: Green Fluorescent Protein
HPRT: Hypoxanthine Guanine Phosphoribosyltransferase
PCR: Polymerase Chain Reaction
T7E1: T7 Endonuclease 1
TNFα: Tumor Necrosis Factor Alpha
ECM: Extracellular Matrix
GAGs: Glycosaminoglycans
PI: Propidium Iodide
HRP: Horseradish Peroxidase
IκB: Inhibitor of κB
IL-6: Interleukin-6
GAPDH: Glyceraldehyde-3-Phosphate Dehydrogenase
MMP-1: Matrix Metalloproteinase-1
MMP-3: Matrix Metalloproteinase-3
IL-1α: Interleukin-1 Alpha
GMP: Good Manufacturing Practice
