# Supplementary material for: Streamlined, single-step non-viral CRISPR-Cas9 knockout strategy enhances gene editing efficiency in primary human chondrocyte populations
Source: Arthritis Res Ther. 2024 Mar 11;26:66. doi: 10.1186/s13075-024-03294-w (PMC10926593; doi:10.1186/s13075-024-03294-w)
Supplement: Supplementary file 1 — Supplementary Material 1. [file 13075_2024_3294_MOESM1_ESM.docx]

**Supplementary Figures**


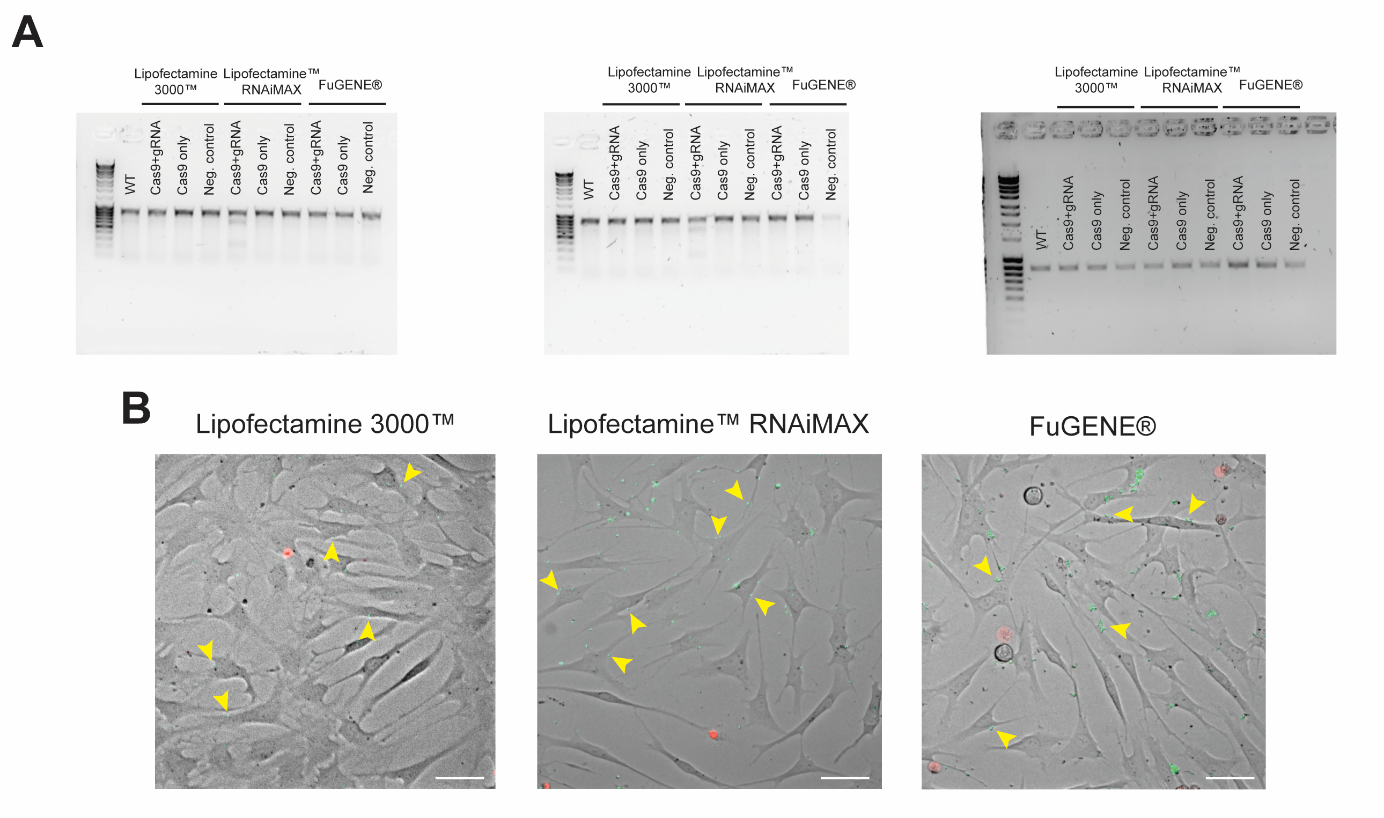


**Supplementary Figure 1**. Cas9-GFP delivery through lipid nanoparticles leads to poor transfection and editing efficiency. (**A**) 1.2% agarose gel runs displaying *HPRT* editing measured by a T7E1 assay of cells transfected with LNPs. (**B**) Representative brightfield microscopy pictures of polydactyly chondrocytes transfected with Cas9-GFP using three different LNPs. Yellow arrows point at GFP^+^ vesicle clusters. Green: Cas9-GFP. Red: Propidium iodide. Scale bars: 50 µm. WT: wild type.


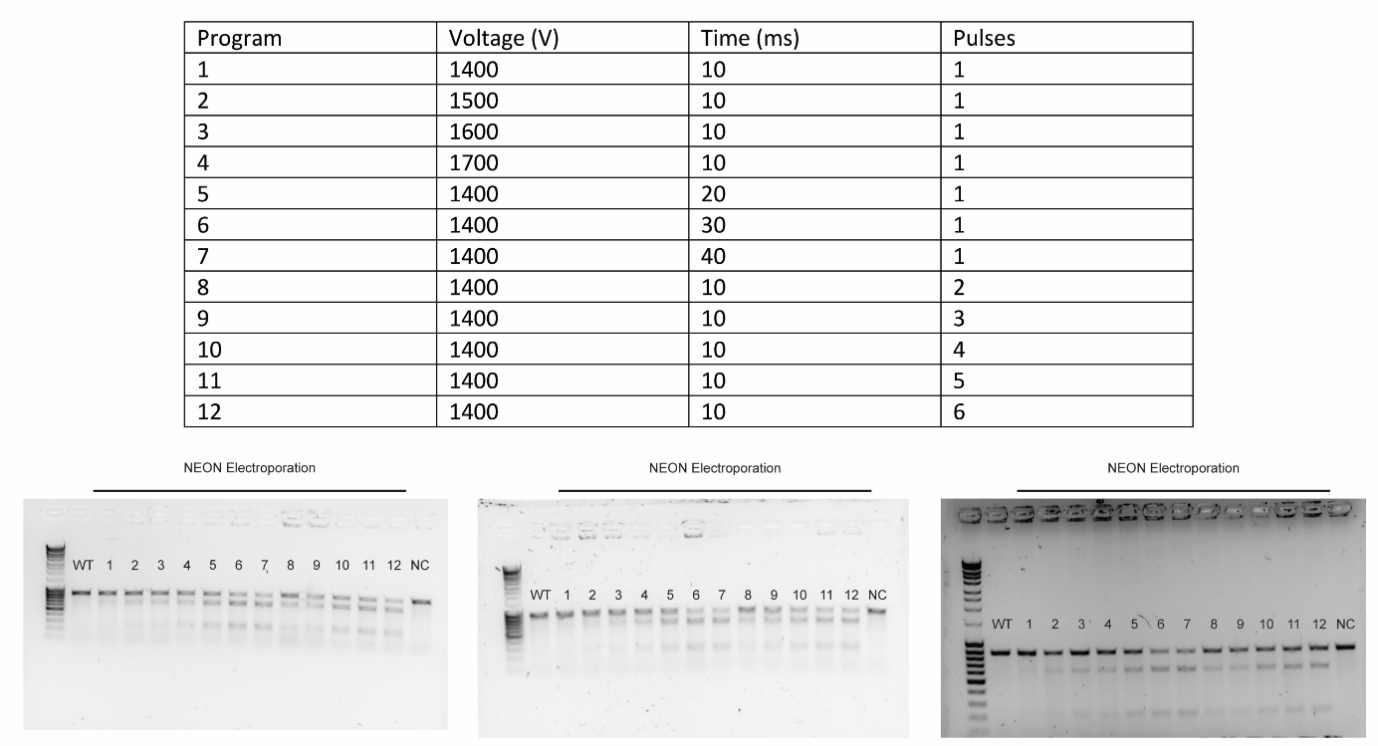


**Supplementary Figure 2**. Increasing voltage, milliseconds, and pulses of the Neon™ electroporator leads to increasingly higher editing efficiency. 1.2% agarose gel runs displaying *HPRT* editing measured by a T7E1 assay of cells transfected with different electroporation programs, as indicated in the table. WT: wild type. NC: negative control (i.e., cells transfected with Cas9 only).


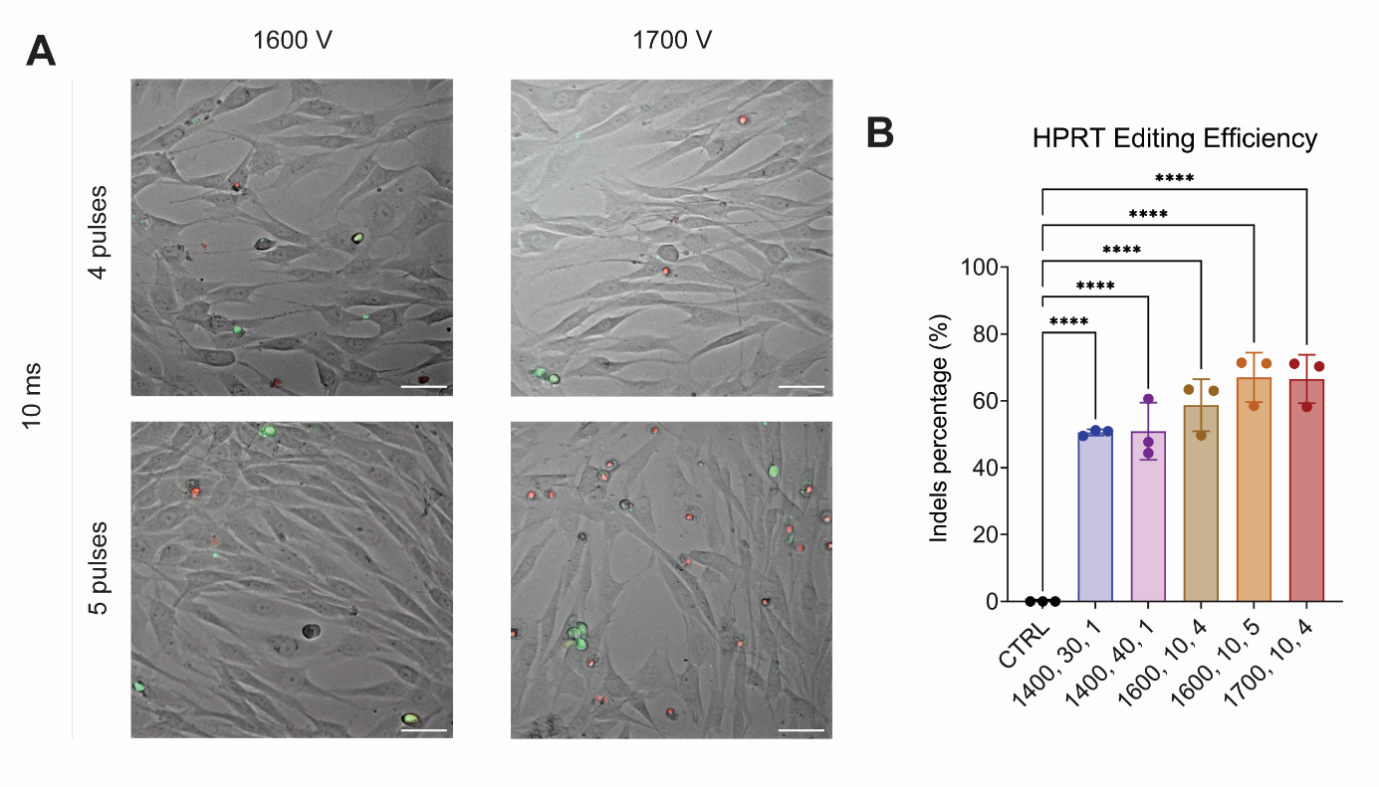


**Supplementary Figure 3**. Electroporation at 1600 V for 5 pulses of 10 ms each maximizes *HPRT* editing efficiency. (**A**) Representative brightfield microscopy pictures of polydactyly chondrocytes transfected with a Cas9-GFP with our final electroporation conditions. Green: Cas9-GFP^+^ cells. Red: Propidium iodide. Scale bars: 50 µm. (**B**) Editing efficiency at the *HPRT* locus for our final electroporation conditions, measured by Sanger sequencing readout. Data are represented as mean ± standard deviation of 3 biological replicates from one donor (n = 3). Statistical significance was determined using one-way ANOVA with a Tukey’s multiple comparisons correction (∗p < 0.05, ∗∗p < 0.01, ∗∗∗p < 0.001, and ∗∗∗∗p < 0.0001).


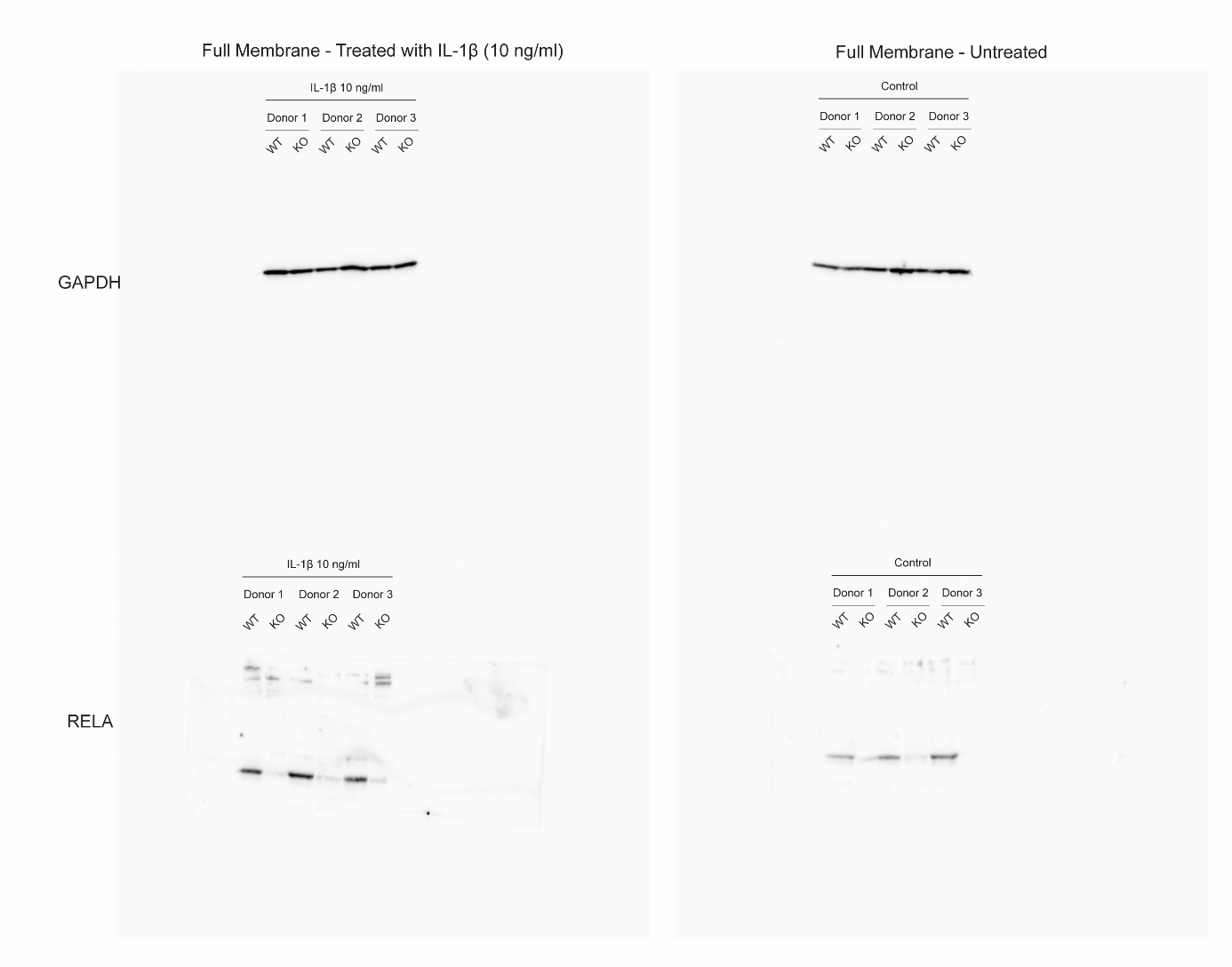


**Supplementary Figure 4**. Full Western blot membranes displaying GAPDH (~37 kDa) and RELA (~ 69 kDa) bands of samples in either an untreated control condition, or treated for 30 minutes with IL-1β at a concentration of 10 ng/ml.


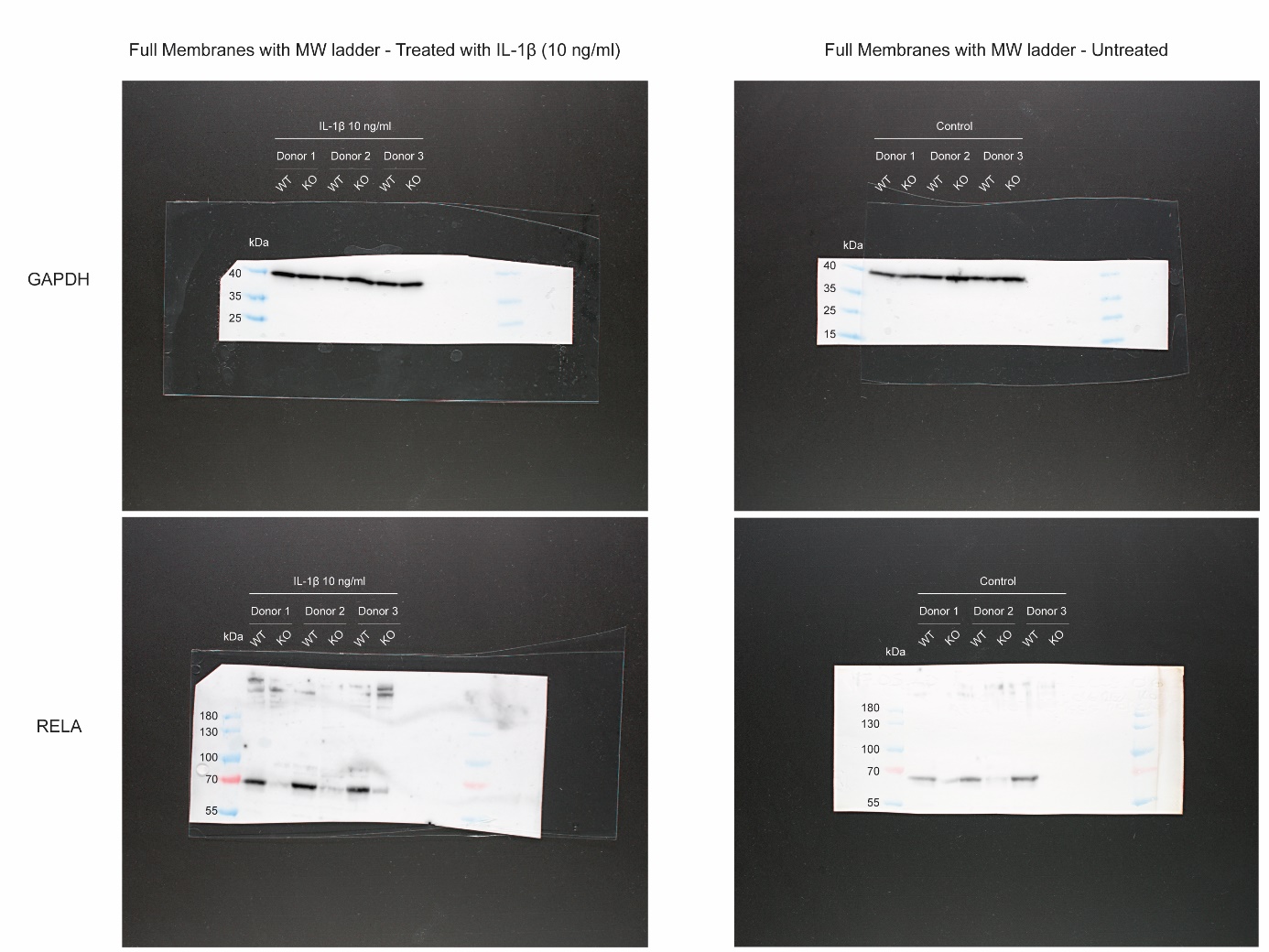


**Supplementary Figure 5**. Full Western blot membranes displaying GAPDH (~37 kDa) and RELA (~ 69 kDa) bands, overlayed with the PageRuler™ Prestained Protein Ladder, 10 to 180 kDa. Samples were either left untreated, or treated for 30 minutes with IL-1β at a concentration of 10 ng/ml.


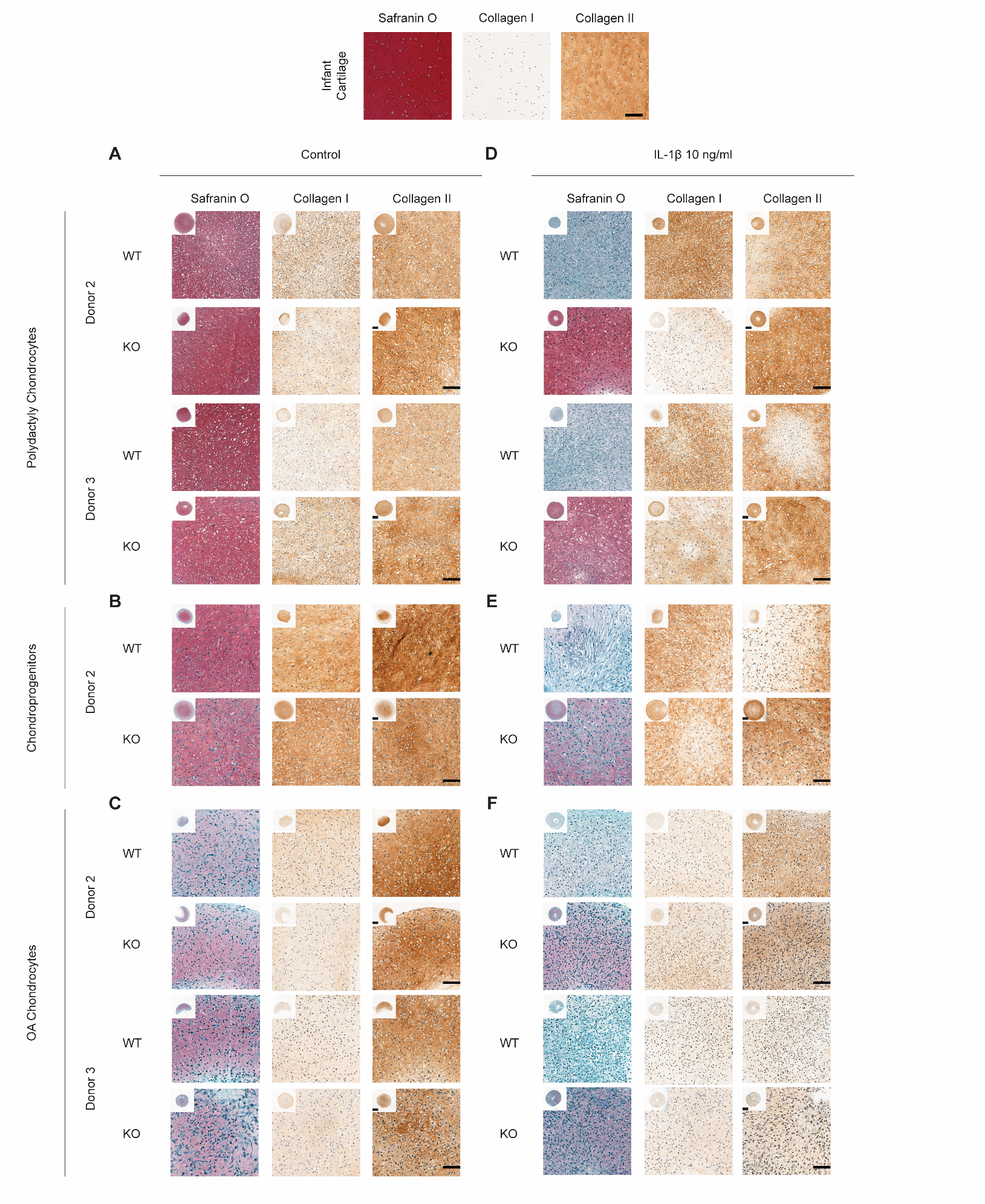


**Supplementary Figure 6**. *RELA* KO chondrocyte pellets display ECM deposition compared to WT cells, and it is enhanced in an inflamed environment. Safranin O, collagen type I and II immunostaining of (**A**) polydactyly chondrocytes, (**B**) FE002 chondroprogenitor cells, and (**C**) OA chondrocyte pellets in an untreated condition after 21 days of culture in chondrogenic media. Safranin O, collagen type I and II immunostaining of (**D**) polydactyly chondrocytes, (**E**) FE002 chondroprogenitor cells and (**F**) OA chondrocytes treated with 10 ng/ml of IL-1β in the last week of culture in chondrogenic media. 20X pictures scale bar, 100 µm; low magnification 2X scale bar, 500 µm.


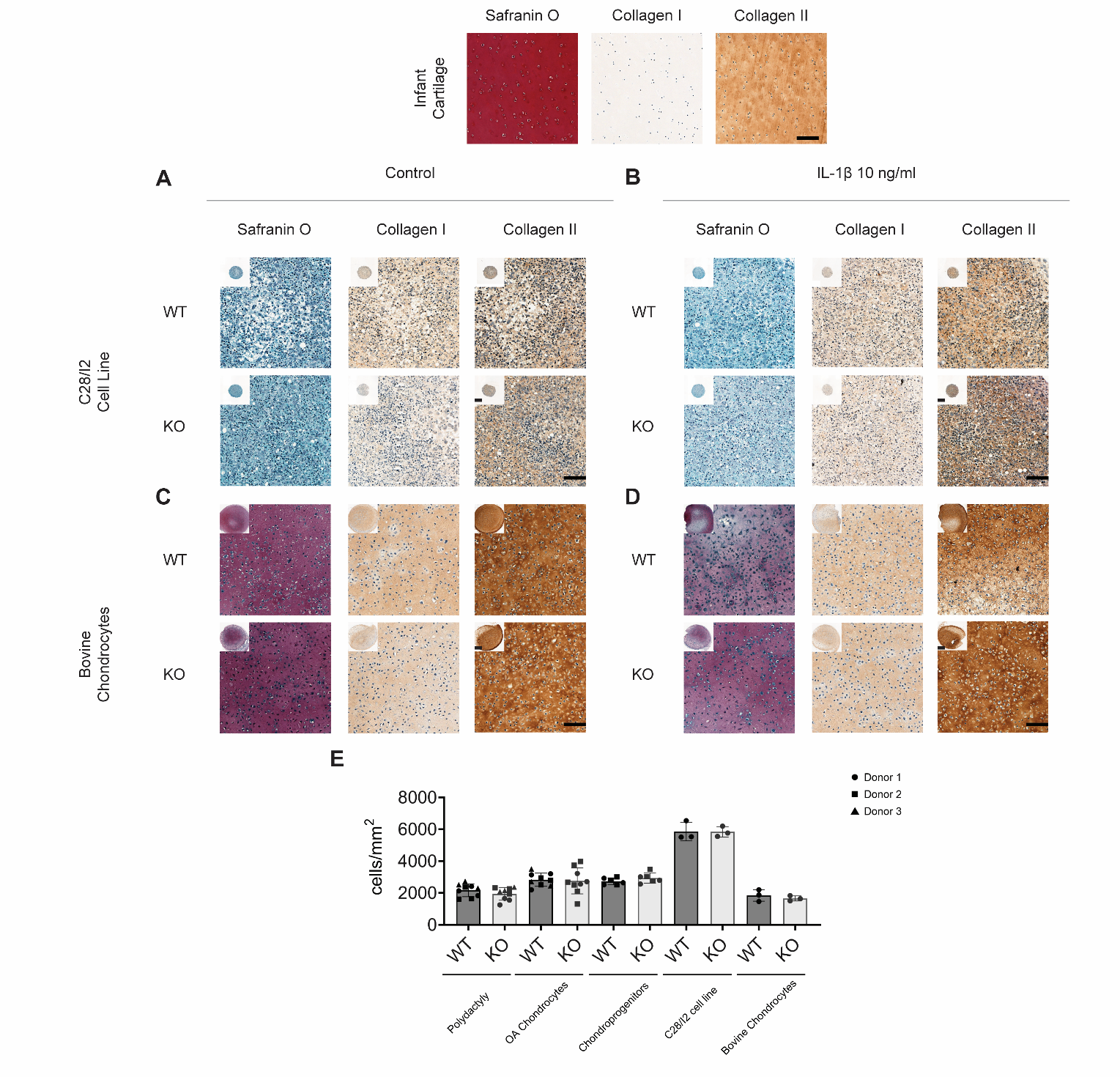


**Supplementary Figure 7**. *RELA* KO cell line and bovine chondrocyte pellets display ECM deposition compared to WT cells, and it is unchanged in an inflammatory environment. Safranin O, collagen type I and II immunostaining of C28/I2 cell pellets in a (**A**) control untreated condition and (**B**) after one week of treatment with 10 ng/ml of IL-1β in chondrogenic media. Safranin O, collagen type I and II immunostaining of bovine cell pellets in a (**C**) control untreated condition and (**D**) after one week of treatment with 10 ng/ml of IL-1β in chondrogenic media. 20X pictures scale bar, 100 µm; low magnification 2X scale bar, 500 µm. (**E**) Untreated pellets number of cells/mm^2^ counted after three weeks of culture in chondrogenic medium. Data is presented as mean ± standard deviation of 3 region of interest (ROI) per pellet, per donor.

**Supplementary Tables**

**Supplementary Table 1**. RELA sgRNAs sequences used in this study, alongside predicted on- and off-target scores as calculated by IDT and the targeted exon of the protein. RELA sgRNAs #1, #2, #4 were designed using the IDT design webtool ([Custom Alt-R™ CRISPR-Cas9 guide RNA | IDT (idtdna.com)](https://eu.idtdna.com/site/order/designtool/index/CRISPR_CUSTOM)). sgRNA #5 was designed with the ChopChop webtool ([CHOPCHOP (uib.no)](http://chopchop.cbu.uib.no/)), while sgRNA #3 was derived from the CRISPRevolution Controls Kit from Synthego. All sgRNAs were ordered from Synthego. For both on- and off-target scores, the higher the score, the better.

| ID | Sequence 5’-3’ | On-target score | Off-target score | Target exon |
| --- | --- | --- | --- | --- |
| RELA sgRNA #1 | TGCTCAATGATCTCCACATA | 73 | 49 | Exon 3 |
| RELA sgRNA #2 | CTGCTCAATGATCTCCACAT | 81 | 39 | Exon 3 |
| RELA sgRNA #3 | GATCTCCACATAGGGGCCAG | 40 | 41 | Exon 3 |
| RELA sgRNA #4 | TCACCAAGGACCCTCCTCAC | 96 | 38 | Exon 4 |
| RELA sgRNA #5 | TCAATGGCTACACAGGACCA | 72 | 36 | Exon 4 |
| Bovine RELA | GGCAAGTACTGGAATTCCAT | 94 | N/A | Exon 8 |

**Supplementary Table 2**. HPRT and RELA genotyping and Sanger sequencing primer sequences used in this study.

| Target | Fwd primer (5’-3’) | Rev primer (5’-3’) | Sequencing primer (5’-3’) |
| --- | --- | --- | --- |
| HPRT | GATGCTCACCTCTCCCACAC | GCCAGGTTCCAGTTCTAAGGA | AGAGAGGCACATTTGCCAGT |
| RELA exon 3 | CTCCGTGGAGAGCAGATTCC | AGTGCTGACCTTGATGGTGG | AGTGCTGACCTTGATGGTGG |
| RELA exon 4 | TGGCCCCTATGTGGAGATCA | CTGAGTCAGGGCAAGGAGTG | TGGCCCCTATGTGGAGATCA |
| Bovine RELA | GCAGAAAGGTACACAGCCAGA | CCCCAGCTTCTGACCTTCA | CCCCAGCTTCTGACCTTCA |

**Supplementary Table 3**. *In silico* predicted off-target genomic locations, sequences, PAM and number of mismatches for RELA sgRNAs #1, #2 and #5 using the [Custom Alt-R™ CRISPR-Cas9 guide RNA | IDT (idtdna.com)](https://eu.idtdna.com/site/order/designtool/index/CRISPR_CUSTOM). Primer sequences for off-target locations are listed. Mismatches are marked in bold.

| ID | sgRNA sequence | PAM | Mismatches | Gene | Score | Location | Fwd (5’-3’) | Rev (5’-3’) |
| --- | --- | --- | --- | --- | --- | --- | --- | --- |
| sgRNA #1 | TGCTCAATGATCTCCACATA | GGG |  | RELA |  | chr11:+65662045 |  |  |
| OT1 | T**CT**TCAA**G**GATCTCCACATA | AGG | 3 | - | 3 | chr14:+83162318 | AGTTCCCGTAGTAGATACACAGC | CCCAGCCTCACTTGAACTCTT |
| OT2 | TG**T**TC**T**AATG**T**TCTCCACATA | TAG | 3 | - | 15 | chr2:-85012958 | ACCACAACTCTTTTCCGCCA | GAGCACTTGCAGTACCCACT |
| OT3 | T**C**CT**T**AATG**-**TCTCCACATA | GGG | 3 | - | 22 | chr18:+40152421 | GCTTGCTTTGGATGTTTTGTGTC | TATTGTGTTGCACTCCAAAGCC |
| OT4 | TGCT**A**AA**G**G**C**ATCTCCACATA | TAG | 3 | - | 22 | chr12:+12865355 | GGGAAACGAAACCAATGAGGC | GCACCTACTTCCTCACCAAGG |
| OT5 | TG**T**TCA**--**GATCTCCACATA | AGG | 3 | - | 26 | chr6:-38176673 | CCTGGTGTGTCCACTCTGTTT | CCTGGGTGTGGAAGAAGGAAC |
| sgRNA #2 | CTGCTCAATGATCTCCACAT | AGG |  | RELA |  | chr11:+65662044 |  |  |
| OT1 | **T**T**C**CTCA**G**ATGATCTCCACAT | CAG | 3 | - | 5 | chr21:+14096613 | TTCCCACAAGCCCCTTTACC | TGCCAGCCACTTTCTAAGGC |
| OT2 | **T**T**C**CTCA**G**ATGATCTCCACAT | CAG | 3 | - | 5 | chr12:+34234721 | GACATAATTCGAGCAGCTGGG | TCAAACACTGCATAGGGGAG |
| OT3 | **T**T**C**CTCA**G**ATGATCTCCACAT | CAG | 3 | - | 5 | chr2:+38710093 | TGACTCTTCTGAGCCCGACT | GAGTGCTGGCAGAATGCAGA |
| OT4 | **G**T**C**CTCA**G**TGA**C**CTCCACAT | TGG | 4 | - | 7 | chrX:+6923032 | TCTTGAGCCCTCTTCATCCTC | AACCGTGAGGCTGAATGAGACA |
| OT5 | **T**TG**T**TC**TT**TGATCTCCACAT | GAG | 4 | - | 21 | chr3:+115018454 | GCCACCAAGGAAAAACAGTGA | TGTTGGTCAGTGTTGTTCCAC |
| sgRNA #5 | TCAATGGCTACACAGGACCA | GGG |  | RELA |  | chr11:-65661814 |  |  |
| OT1 | T**T**A**G**TG**A**CTACACAGGACCA | GGG | 3 | - | 2 | chr16:-55390144 | TGAGAACCCGATGACTGGTG | TCCAGATCTCGGCCATCAAAC |
| OT2 | TCA**C**T**CA**CTACACAGGACCA | AGG | 3 | - | 11 | chr12:-129637475 | CCTACATCACCCTACAACTGC | AGGCTTTACAGGAAGCATGGC |
| OT3 | T**A**AA**G**G**AA**TACACAGGACCA | CAG | 4 | - | 19 | chr3:+77775444 | TAACGCAGGTGGATGGCAAAT | ACATAACAGCAGGCAACTCCT |
| OT4 | **A**CAATG**TTC**ACACAGGACCA | GGG | 4 | - | 20 | chr17:-14116917 | ACGCAGGACACCACAAGTTTA | AGCATGGTGAACATAAGCCCA |
| OT5 | T**A**AATG**A**CTA**T**CACAGGACCA | GAG | 3 | - | 24 | chrX:-6647627 | AGTTGAAGTCAGCATCGGGG | CTGATCTTGAACTCCTGACCTC |

**Supplementary Table 4**. RT-qPCR primer sequences used in this study.

| Target | Fwd primer (5’-3’) | Rev primer (5’-3’) |
| --- | --- | --- |
| GAPDH | AGTCAGCCGCATCTTCTTTT | CCAATACGACCAAATCCGTTG |
| MMP-13 | CCTTCAAAGTTTGGTCCGATG | TCAAATGGGTAGAAGTCGCC |
| IL-1β | CTGAGCTCGCCAGTGAAATG | TCTGTTTAGGGCCATCAGCTT |
| IL-6 | GAAAGCAGCAAAGAGGCACT | TTTCACCAGGCAAGTCTCCT |
| TNF-α | ATCCTGGGGGACCCAATGTA | AAAAGAAGGCACAGAGGCCA |
